# Seismic assessment of bridges through structural health monitoring: a state-of-the-art review

**DOI:** 10.1007/s10518-023-01819-3

**Published:** 2023-11-30

**Authors:** Christos Karakostas, Giuseppe Quaranta, Eleni Chatzi, Abdullah Can Zülfikar, Oğuzhan Çetindemir, Guido De Roeck, Michael Döhler, Maria Pina Limongelli, Geert Lombaert, Nurdan Memişoğlu Apaydın, Vikram Pakrashi, Costas Papadimitriou, Ali Yeşilyurt

**Affiliations:** 1 Institute of Engineering Seismology and Earthquake Engineering, Research Unit of Earthquake Planning and Protection Organization, Thessaloniki, Greece; 2 Department of Structural and Geotechnical Engineering, Sapienza University of Rome, Rome, Italy; 3 Department of Civil, Environmental and Geomatic Engineering, ETH Zürich, Zurich, Switzerland; 4 Disaster Management Institute, Istanbul Technical University, Istanbul, Türkiye; 5 Department of Civil Engineering, Gebze Technical University, Kocaeli, Türkiye; 6 Department of Civil Engineering, Katholieke Universiteit Leuven, Leuven, Belgium; 7 Université Gustave Eiffel, Inria, COSYS-SII, I4S, Rennes, France; 8 Department of Architecture, Built Environment and Construction Engineering, Politecnico di Milano, Milan, Italy; 9 Department of Civil Engineering, Istanbul University, Istanbul, Türkiye; 10 UCD Centre for Mechanics, Dynamical Systems and Risk Laboratory, School of Mechanical and Materials Engineering, University College Dublin, Dublin, Ireland; 11 Department of Mechanical Engineering, University of Thessaly, Volos, Greece

**Keywords:** Bridge, Computational intelligence, Condition assessment, Damage detection, Identification, Fatih Sultan Mehmet bridge, Optimal sensor placement, Seismic assessment, Structural health monitoring, Suspension bridge

## Abstract

The present work offers a comprehensive overview of methods related to condition assessment of bridges through Structural Health Monitoring (SHM) procedures, with a particular interest on aspects of seismic assessment. Established techniques pertaining to different levels of the SHM hierarchy, reflecting increasing detail and complexity, are first outlined. A significant portion of this review work is then devoted to the overview of computational intelligence schemes across various aspects of bridge condition assessment, including sensor placement and health tracking. The paper concludes with illustrative examples of two long-span suspension bridges, in which several instrumentation aspects and assessments of seismic response issues are discussed.

## Introduction

Bridges form critical infrastructure components of high financial and societal relevance. As evidenced by recent catastrophic events (Morgese et al. 2020), in many, predominantly developed countries a number of these systems are reaching their end of life, or are faced with severe deterioration, rendering them more prone to damage from extreme actions, such as seismic loading. For ensuring the safety and efficient operation of these systems, a regular procedure for inspection and maintenance is mandated by current codes and standards, relying primarily on use of visual inspections as a tool for condition assessment. While valuable, this type of assessment is typically periodic (taking place every 3–5 years) and relies on detection of flaws that have visually manifested, often expressing an advanced state of damage (cracking, spalling, loss of joint capacity, corrosion). If such an advanced damage state is suspected, reassessment of the system capacity is necessary, and could be supplemented with information acquired through destructive (e.g., extraction of specimens for lab testing) or non-destructive testing (e.g., ultrasonic, acoustic emission, impact-echo, ground penetrating radar, etc.). Such non-destructive evaluation (NDE) procedures can be extremely efficient in obtaining information that can more reliably be used to set up informed models of structural conditions, but remain more or less sporadic in the manner in which they may be applied.

**Fig. 1:**
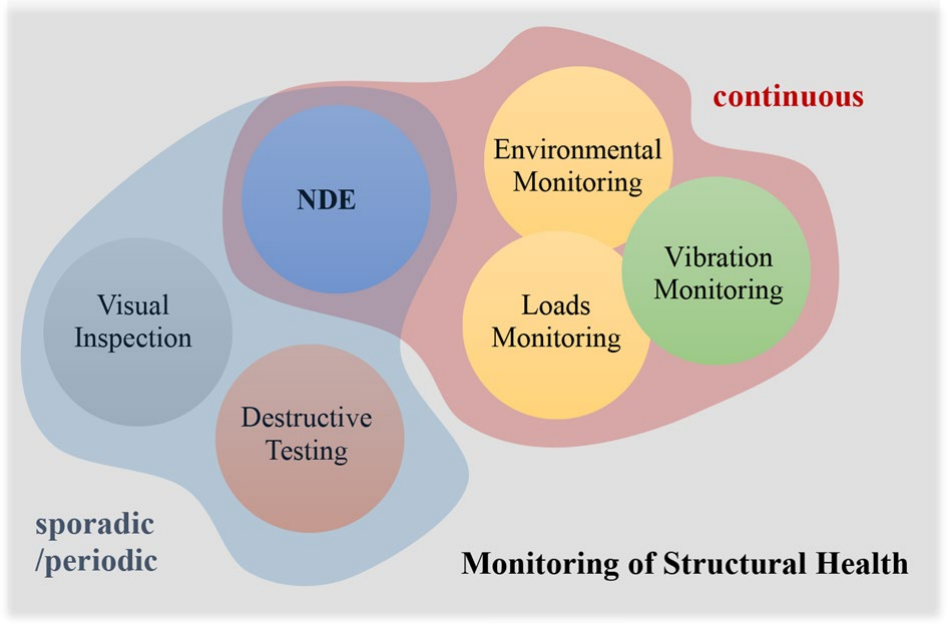
The variants of monitoring of structural health in terms of sporadically and continuously deployed regimes, adapted from (Ou 2020)

As an alternative to the sporadic approach to information extraction on structural health, long-term continuous Structural Health Monitoring (SHM) systems can be deployed, which provide a supervision of the system throughout its complete operational spectrum. Continuous monitoring often relies on vibration-based monitoring, which track dynamic response quantities, such as displacements, accelerations, tilts and strains, as well as operational (e.g., traffic) and environmental variables (e.g., wind, temperature and humidity). Despite this separation, sporadic/periodic and continuous implementations of monitoring, including classical (e.g., visual) inspection systems, fall under the broad umbrella of monitoring of structural health, as summarized in Fig. 1. An up-to-date state of the art on several aspects of seismic SHM is contained in (Limongelli and Celebi 2019). In this work, we will be focusing on continuous monitoring approaches for seismic assessment of bridges, organized across the four main objectives of SHM, as identified by Rytter (1993), namely damage detection, localization, quantification, and residual life prediction.

SHM leverages on continual developments in sensing, data acquisition and transmission systems, as well as processing and analysis modalities to assess structural condition for the purposes of structural characterization, design verification, operation and maintenance decision-support, and post-event management (Brownjohn 2007; Farrar and Worden 2012). The increasing availability of low-cost (typically MEMS-based) and easily deployable sensing solutions promotes the development of so-called self-aware structures, which are equipped with dense sensing deployments and feature supervisory systems which support partly autonomous supervision (Adam and Smith 2006; O’Connor et al. 2014; Yao and Glisic 2015; Laflamme et al. 2016). In the context of SHM for seismic monitoring of bridges, dynamic response measurements, acquired through suitable sensor networks, are used to localize and quantify local damage and degradation of structural elements or anti-seismic devices (Graham and Campbell 2013). Seismic monitoring of structures (Fujino et al. 2019; Martakis et al. 2019; Zhang et al. 2023) in particular aims to (i) assess the seismic performance of bridge systems, (ii) identify bridge properties and compare these against design assumptions, and (iii) capitalize on gained knowledge to improve future designs and practices (An et al. 2019; Rizzo and Enshaeian 2021).

Key to the SHM procedure is the post-processing step pertaining to interpretation of the acquired information into identification of the structural/dynamic properties of the monitored system, as well as inference of indicators of its performance. The information, which remains latent in the data, is often coupled with physics-based models, to update our knowledge of the as-is system, leading to updated numerical representations; also referred to as digital twins (Martakis et al. 2022). Alternatively, a purely data-driven approach may be followed for the extraction of condition/performance indicators (Noh et al. 2011).

A number of review works have been delivered over the past decades reporting on the evolution of SHM approaches for damage detection, which comprises one of the primary tasks of SHM, including the works by Doebling et al. (1996); Salawu (1997); Chang et al. (2003); Sohn et al. (2003); Fan and Qiao (2011); Seo et al. (2016). To what bridge systems in particular are concerned, the interested reader is referred to the review works of Sun et al. (2017); Casas and Moughty (2017), and more recently An et al. (2019), which however do not focus on seismic actions. Seismic monitoring, at large, is discussed in (Limongelli and Çelebi 2019), with one of the sections dedicated to bridge studies in British Columbia (Kaya and Ventura 2019). In Limongelli et al. (2018), the link to performance indicators is made, while Prendergast et al. (2018) describe monitoring under multiple natural hazard effects, namely for the case of flooding and seismic actions.

In this paper, we offer a review of works relating to monitoring schemes, which have been applied, or bear application potential, for use in seismic health monitoring of bridges. Since latent phenomena can influence the seismic behavior of the bridges, short notes are also included about the structural monitoring of the effects due to aging, deterioration and fatigue. Sections 2–4 give a comprehensive review on available methodologies for various levels of damage detection with increasing detail of in-depth information i.e. simply detecting the appearance of damage in a bridge (Level 1—Damage detection), additionally detecting the location(s) of damage (Level 2—Localization), assessing the degree of damage (Level 3—Quantification) and finally evaluating the residual life of a damaged bridge (Level 4—Residual damage prediction). A significant contribution of the present work is the comprehensive presentation in Sect. 5 of the use of Computational Intelligence in all aspects of structural monitoring i.e. optimal sensors locations, system identification, model updating and all levels of damage detection issues. Although, as mentioned above, the presented methodologies primarily focus on seismic health monitoring issues, the role of other environmental and operational factors affecting the dynamic behaviour of a bridge are also partially mentioned. Finally, in Sect. 6, illustrative examples of the monitoring aspects of two long suspension bridges over the Bosporus channel are presented in detail. Some basic analyses based on recorded data are also performed, mainly in order to present some important aspects of the procedure for the assessment of the structural health of one of the bridges due to a low-intensity seismic event. In order to facilitate the reader, a list of acronyms used throughout the text is presented in Appendix.

## Damage detection (level 1)

When concerned with the 1st level of SHM assessment, i.e., the task of detection, the majority of methods available in the literature may be classified into data-driven or model-based approaches (Moallemi et al. 2021). Data-driven methods rely on the inference of damage-sensitive features without exploiting availability of a physics-based system model (Azimi et al. 2020). Model-based methods, on the other hand, assume availability of a system model, most typically numerical (e.g., finite-element-based), which is often updated to match the acquired measurements (Ghahari et al. 2022). For the task of detection, purely data-driven methods prove highly efficient. However, lack of physical connotation hinders the more advanced tasks of the SHM hierarchy, such as quantification and localization. Model-based methods are better suited for such tasks, albeit coming with significantly higher computational costs, which hinders their implementation in real-time applications (e.g., real-time diagnostics and control). Hybrid approaches also exist, where finite element (FE) information is injected into data-driven methods in order to yield damage indicators that can exploit model-based features, without the need for model updating (Mendler et al. 2021). Below follows an overview of utilization of the aforementioned methodologies for damage detection.

### Data-driven methods

In the typical implementation of data-driven approaches, we seek to identify distinctive features, with the extent of the discrepancy between the reference (healthy), and inspected configuration revealing damage. This discrepancy is typically quantified in terms of statistical metrics, with a relevant threshold also prescribed. As reported by Limongelli et al. (2016), data-driven methods may be further categorized on the basis of their featured signal processing tools. In the SHM context, we distinguish frequency-based methods, time series method and time-variant methods.

#### Frequency-based methods

In time-invariant frequency-based methods, some form of spectral analysis (e.g., Fourier transform, frequency domain decomposition method, complex mode indicator function) is employed as the signal-processing tool for extraction of quantities that primarily relate to the modal characteristics of the system (i.e., frequencies, mode-shapes and damping), which are typically extracted through the process of operational modal analysis (OMA) (Reynders 2012; Cabboi et al. 2017), under ambient excitation conditions. When it comes to seismic assessment, the features identified prior to and after a seismic event may be contrasted for the purpose of damage detection. This is reported as early as 1991 in the work of Hearn and Testa (1991) in laboratory structures, and was later extensively demonstrated on operating systems, most notably on the Z24 bridge benchmark (De Roeck and Maeck 1999; Maeck and De Roeck 2003; Reynders and Roeck 2009). The Z24 bridge was an operating field structure in Switzerland, which was monitored under a 9-month period under ambient conditions, and subsequently controllably damaged. A number of works have been published in recent years demonstrating successful damage detection on the basis of tracking of modal properties of a bridge, but also underlining that natural frequencies are not always sensitive to local damage (Farrar and Doebling 1999), and stressing the importance of including information on strain, which is better linked to local damage effects (Deraemaeker 2010; Pakrashi et al. 2010, 2013). The need of reducing the data transmission payloads in wireless monitoring networks also originated some compressive sensing based approaches relevant for time-invariant frequency-based SHM methods (Bao et al. 2011; Gkoktsi et al. 2019). For instance, Gkoktsi and Giaralis (2020) proposed a novel compressive sensing approach able to detect seismic-induced structural damage associated with changes to natural frequencies as minor as 1% by sampling up to 78% below the Nyquist rate.

Perhaps the most challenging aspect in purely data-driven damage detection lies in the influence of environmental and operational parameters (EOPs). Farrar et al. (1996) observed a 5% variation in the natural frequencies of the Alamosa Canyon Bridge within a 24-hour period due to spatial temperature gradients. Alampalli (2000) reports a 50% variability for a bridge in Claverack (NY) due to freezing of the supports. A 14–18% variability is also reported for the case of the Z24 bridge benchmark (Reynders and Roeck 2009). For decoupling such operational (and therefore benign) effects from structural damage, statistical methods are commonly exploited. These may be distinguished in two main classes, namely output-only methods, which assume no direct measurement of the environmental inputs (e.g., temperature) (Kullaa 2011), and input–output methods where a relationship is inferred between measured EOPs and monitored response features (e.g., frequencies) (Peeters and De Roeck 2001). As an example of the first method, Reynders et al. (2014) applied kernel Principal Component Analysis (k-PCA), for extracting a condition indicator by eliminating environmental and operational influences on natural frequencies. In recent work, cointegration techniques have been utilized to remove EOP effects on the modal frequency set (Cross and Worden 2012; Worden et al. 2014). In following the second path (input–output), Spiridonakos and Chatzi (2014) employ Polynomial Chaos Expansion (PCE) to derive a functional representation between inputs and outputs, delivering a robust performance indicator, verified on damage detection for the Z24 bridge. A detailed assessment of feature extraction and assessment of Z24 and S101 bridge in terms of time and frequency aspects has recently been carried out Buckley et al. (2022). As an alternative to modal information, flexibility-based methods may also be used, as demonstrated in the works by Schommer et al. (2017a) and Nobahari and Seyedpoor (2013). The flexibility matrix may be easily extracted from dynamic response measurements, however, when compared against stiffness information, it is less straightforwardly linked to damage quantification and localization.

The condition/damage indicators are typically constructed on the basis of discrepancies between measured and estimated quantities. However, the task of detection is not finalized upon mere extraction of a performance indicator. In addition to this, robust outlier analysis has to be implemented as demonstrated in the work of Dervilis et al. (2015). Tools such as robust regression analysis, relying on least trimmed square and minimum covariance determinant estimators, can assist in further clustering the outlier space, so as to deem those outliers which may reliably serve as warnings, against possible false alarms. This work was validated on monitoring data from the Z24 and Tamar Bridges, and would be suitable for detection also in the case of damage inflected from seismic events. Further options for statistical analysis for robust damage detection are overviewed in the work by Comanducci et al. (2016), where detection based on novelty, multiple linear regression and a linear Principal Component Analysis (PCA) is discussed.

#### Time-domain methods

Time domain information can be exploited in raw form for the purpose of damage detection. Condition indicators that are inferred on the basis of field measurements have long been adopted by Caltrans to characterize post-earthquake bridge response. This includes markers at expansion joints to track bridge movements or tension indicators on cable restrainers to indicate yielding of the cables (Chen and Duan 2014). Particularly to what concerns bridges involving cable elements (cable-stayed, suspension), the monitoring of tension forces in the stay cables, by direct (e.g., load cell) or indirect (e.g., vibration-based tension estimation) methods, translates to a direct indicator for relaxation losses, strength reduction and triggering of remedial action (Mazzeo et al. 2023; Scarella et al. 2017).

Beyond exploitation of signals as is, generic dynamic monitoring data may be fed into indirect estimation methods for the purposes of identification and detection. Time-domain methods typically pertain to the use of time-series analysis tools, in the form of state-space [e.g., subspace system identification-based (Döhler et al. 2014; Greś et al. 2021)] or autoregressive-type models, for describing the monitored response in an input–output or output-only setting, with single or multiple inputs/outputs. Such parametric methods may be further classified into model parameter-based methods, residual-based methods, and functional model-based methods. The classification depends on whether fault detection and identification rely on (i) direct use of the parameter vector of a time series model, or (ii) functions of residual sequences obtained by driving the current signal(s) through suitable pre-determined models estimated in a baseline phase, or (iii) a combination of the two. The interested reader is referred to the work by Fassois and Sakellariou (2007) for a detailed overview of these methods. The application of statistical damage detection with the subspace-based residual, as well as frequency and mode shape-based detection to the case study of an extensive progressive damage test of a prestressed concrete bridge, namely the S101 Bridge, is reported in Döhler et al. (2014); Greś et al. (2021b). Monitoring of an impact damaged bridge throughout repair has been carried out via a dynamic harmonic approach Buckley et al. (2021b). Changes in estimated extreme value fits to earthquake responses have also been linked to detection of damage (Pakrashi et al. 2018).

#### Time-variant methods

While the former tools are most commonly adopted in the context of stationary loads, time-variant methods are particularly relevant when dealing with non-stationary loads, such as seismic loading. These include time-series models featuring time-dependent coefficients, such as linear parameters varying time series models (Avendaño-Valencia et al. 2017), time varying autoregressive models (Bogoevska et al. 2017), or functionally pooled time series models (Kopsaftopoulos et al. 2018), time-frequency methods that analyse time variations of the spectral quantities using, for example, the Wigner–Ville distribution and time-scale methods that decompose the signal based on a priori chosen functions, e.g. wavelets. A review of these methods can be found in (Staszewski and Robertson 2007).

Wavelet-based approaches also fall in this class of methods (Spiridonakos and Chatzi 2013; Hera and Hou 2004; Nair and Kiremidjian 2009; Pakrashi et al. 2007). In this context, Noh et al. (2011) proposed engineering demand parameter indicators for non-model-based seismic vulnerability assessment of steel frame structures, while Hwang and Lignos (2017) demonstrated that wavelet-based damage-sensitive features can facilitate seismic vulnerability assessment. These rely on tracking of damage sensitive features inferred from wavelet analysis. As an example of such an analysis, Golmohamadi et al. (2012) calculate statistical moments of the energy density function of monitored signals and detect damage on the basis of discrepancies of the wavelet coefficients prior to and after damage. This work is verified on a numerical model of a full-scale railway bridge. The interested reader is pointed to (Kankanamge et al. 2020) for a review on applications of the continuous wavelet transform in SHM.

### Model-based methods

In model-based methods, damage is typically identified through the updating of a FE model (Simoen et al. 2015; Jia et al. 2022; Argyris et al. 2020). The basic premise is that structural damage results in a reduction of stiffness, which synchronously leads to the further tasks of localization and quantification, as elaborated upon in Sect. 3. Model-based schemes are particularly useful for moving beyond the task of diagnosis of new or established damage to the prognostic tasks of reliability and remaining useful life assessment.

## Damage localization and quantification (levels 2 and 3)

The more advanced tasks of damage localization and quantification may be succeeded, as in Level 1, by means of purely data-driven or model-based schemes, however, we here additionally refer to the so-called hybrid methods which rely on—usually online–interaction of data and models.

### Data-driven methods

Purely data-driven methods come with the handicap of ignoring the physical configuration of the system, with damage localization thus largely dependent upon the spatial arrangement of the network of sensors that is deployed.

#### Mode shape-based methods

Based on the chosen sensor placement, data-related quantities that may indicate location and severity of damage largely rely on utilization of mode shapes, or their derivatives such as mode shape curvatures (MSCs) (Lacarbonara et al. 2016), and associated quantities such as modal strain energy (Zhao et al. 2020; Tatsis et al. 2018) or interpolation error (Limongelli 2010). Khan et al. (2022) proposed a novel approach for estimating modal parameters of bridges, including damage-induced changes of boundary conditions, by using progressively redeploying sensors along the axis of a monitored bridge. Numerical and experimental results demonstrated that mode shape and its gradient can successfully detect a change in support conditions.

In general, lower modes are often less sensitive to local damage and are affected by variation in the EOPs, and their effects onto boundary conditions in particular. On the other hand, higher modes are non-trivial to extract from ambient information, and come with high variance at the cost of reliability. In alleviating dependence on EOPs, Shokrani et al. (2018) extended the PCA-based approach, previously applied onto natural frequencies, for use with MSCs. This led to derivation of EOP-neutral indicators of performance with a spatial identity, and the framework is demonstrated to work on bridge-like structural systems. As an alternative to using modal-shapes, operational deflection shapes may also be employed. In (Giordano and Limongelli 2020), a comparison of damage indices computed with modal and operational shapes is reported for the case of a real bridge. Operational shapes correspond to the deflection of a structure at a particular frequency, and are often easier to experimentally derive (Sampaio et al. 1999; Dilena et al. 2015) even if they might be more affected by noise due to the lower signal to noise ratio with respect to modal shapes.

#### Time-domain methods

Time-domain-based methods pertain to either direct processing of raw measurement time histories, or on time-domain identification methods applied onto monitored signals. As an example of the first, Tondreau and Deraemaeker (2014) introduced an unsupervised technique for damage localization relying on use of dynamic strain measurements, which are passed through so-called “modal filters”. Due to strong linkage between strains and local damage, this method proves more robust than attempts relying on use of accelerations. Similarly, the shift in the position of the neutral axis, obtained via static or dynamic strain measurements can serve as a means for localizing damage. An and Ou (2012) utilize the curvature of the waveforms of the logarithm of mean squares of measured acceleration signals, and employ the discrepancy prior to and after damage as for damage localization.

Particularly to what concerns seismic assessment, quantities which may be estimated via measurements, such as seismic displacements, reinforced concrete pier/column drift ratios and residual displacements (Tesfamariam and Goda 2015; Yazgan and Dazio 2011), plastic rotations (for ductile members), or shear forces at the members/joints (for non-ductile members) can serve as direct indicators of performance (Kolias et al. 2012). Residual drifts form an established indicator of performance in the Japanese seismic design specifications for highway bridges (Kawashima and Unjoh 1997).

Beyond quantities that are readily extracted from measurements, quantities derived as part of time-domain system identification procedures may also serve for the purpose of localization. Subspace identification methods, and the resulting data Hankel matrix, may be used to define a null-space damage index (Loh et al. 2015). The reference data (undamaged data) is utilized as a baseline for calculating the discrepancies driving the formulation of the damage indices. Deviations in the components of the respective indices serve for localization. Such a Hankel matrix based approach has led to the development of near real-time implementation of data detection through an eigenperturbative approach and utilising extreme value estimates to identify significant changes (Bhowmik et al. 2019; Pakrashi et al. 2018).

### Model-based methods

In model-based methods, damage is typically identified through coupling with a FE model, which is updated to reflect the monitored structure (Teughels and De Roeck 2005; Simoen et al. 2015; Martakis et al. 2021). For the purpose of damage localization, a damage index needs to be formulated on the basis of some measure of discrepancy (fitness function). Under availability of a FE model, structural damage is tied to reduction of stiffness, or local change of the modulus of elasticity. Therefore, the variable to be updated typically includes structural parameter information, such as the Young’s modulus of elasticity, or if data from nonlinear tests or seismic response are available, then strength parameters may also enter the set of variables to be identified. In its most basic form, model parameterization pertains to parameterization of the stiffness matrix:

1 $$\begin{aligned} \textbf{K}\left( \theta \right) =\textbf{K}_0 + \sum _{j=1}^N{\theta _j \textbf{K}_j}, \end{aligned}$$

where each $\textbf{K}_j$ is the contribution of a single substructure to the global stiffness matrix and $\theta _j$ is a scaling representative of its effective stiffness.

In case of availability of ambient data, the fitness function expressing the discrepancy between monitored and estimated quantities, typically relies on matching of modal properties, which are extracted from measured response time histories using modal analysis techniques (Reynders 2012). In its most fundamental form, the fitness function may be formulated on the basis of natural frequencies or eigenvalues, although it is further possible to include modal assurance criterion (MAC) values for mode shape, or modal curvature discrepancies (Meruane and Heylen 2012; Quaranta et al. 2016). In presence of diverse components in the fitness function, appropriate weighting may be employed to scale the individual contributions. Many alternative formulations are possible, but most often this is expressed as a weighted least squares fit between predictions and data:

2 $$\begin{aligned} F\left( \varvec{\theta }\right) =\dfrac{1}{2}\varvec{\eta }(\varvec{\theta })^\top {\varvec{W}} \varvec{\eta }(\varvec{\theta }), \end{aligned}$$

where $\varvec{\eta }$ is the vector containing the residuals between predicted and measured data (natural frequencies, mode shapes, etc.) and ${\varvec{W}}$ is a weighting matrix. In most practical applications, a diagonal weighting matrix is chosen, so that the cost function becomes a sum of squared residuals. The optimal value $\varvec{\theta }^*$ of the parameter vector $\varvec{\theta }$ is determined through the solution of a non-linear least-squares problem:

3 $$\begin{aligned} \varvec{\theta }^*= \mathop {\textrm{argmin}}\limits _{\varvec{\theta }} F(\varvec{\theta }). \end{aligned}$$

Local gradient-based optimization methods are most often used to solve the optimization problem. For modal data such as natural frequencies and mode shapes, the gradient can be efficiently calculated analytically, avoiding the use of finite differences. Alternatives relying on adoption of machine learning approaches, such as neural networks (Alavi et al. 2016) or statistical pattern recognition approaches have also been proposed for detection and localization (Betti et al. 2013). Model updating comprises an inverse problem and is often prone to ill-posedness and ill-conditioning. Accounting for uncertainties due to measurement and modeling errors is therefore essential (Simoen et al. 2015), with a Bayesian approach often used to this end (Ntotsios et al. 2009; Figueiredo et al. 2014; Behmanesh and Moaveni 2015).

In particular, the ill-conditioning in the inverse problem of damage identification arises from the potentially large number of model parameters introduced to monitor potential structural changes due to damage in a large number of substructures of the entire structure. However, damage in a structure is expected to occur at a limited number of locations. The prior information that the spatial distribution of damage is sparse can be exploited to reduce ill-conditioning. In deterministic approaches, *l*_1_ regularization techniques are used to enforce sparsity. Applications include load identification of bridges (Bao et al. 2016) and damage identification of structures using measured model properties (Hou et al. 2018). In probabilistic approaches, sparse Bayesian learning techniques (Tipping 2001), introduced originally to obtain sparse solutions to regression and classification problems (Bishop 2006), use automatic relevance determination prior to promote sparsity. They have been successfully applied to damage identification using linear systems with measured modal properties (Huang and Beck 2015, 2018; Hou et al. 2019) and for parameter estimation for sparse spatial distribution of damage in nonlinear structures using measured response time histories (Filippitzis et al. 2022).

As reported in (An et al. 2019), instances of model updating procedures applied for bridge systems include multi-resolution analysis (Zhang et al. 2015b), use of 3D simulation models (Saidou Sanda et al. 2017), exploitation of in-situ tests for prestressed concrete bridges (Schommer et al. 2017b) and fusion of static with dynamic strains, obtained from fiber optical sensors (Wu et al. 2017). In order to reduce the computational cost of the localization problem, Teughels et al. (2002) employ a limited set of damage functions, where the updating parameters are the factors by which each of the damage functions has to be multiplied prior to recombination. The damage functions are determined by means of shape functions, known from FE theory. In an alternate approach to reduction, Papadimitriou and Papadioti (2013) use component mode synthesis, to extract a reduced order representation of the full FE model, and a Bayesian approach for updating parameters in a reduced space of generalized coordinates. The method is demonstrated on simulated data from Metsovo Highway Bridge.

### Hybrid methods

A third group of methods for damage localization and quantification exploit online interaction of data and physics-based models (differently to the offline concept of model updating), within a hybrid scheme.

A representative method of this class is the damage locating vector approach proposed by Bernal (2002). This scheme relies on detection of changes in the structural flexibility in order to localize damage. The method has been extended to output-only data (Bernal 2010), where a vector is estimated in the null space of the transfer matrix difference between reference and damaged states, as these are defined purely on the basis of data. Application of this vector as a virtual load to the FE model of the structure, results in zero stress over the damaged elements. Based on this, a damage indicator may be defined for each structural element, whose value for damage equals zero. To formalize localization, Döhler et al. (2013) employ a statistical test.

Statistical fault isolation methods define a theoretical framework for localization of damage, seeking to specify which parameters of an available numerical (e.g., FE) model have changed under availability of measurements from a baseline and damaged state. Formally, this is different to a model updating procedure, since in a first step only change in the involved parameters is sought by means of statistical hypothesis testing (Allahdadian et al. 2019). Once damage is detected, it can be more precisely linked to localization and even quantified by more carefully examining damaged elements (Döhler et al. 2016; Greś et al. 2021a).

Another class of hybrid methods, exploits availability of a model of the structure, and couples this model with data in order to predict damage in real-time. Bayesian filters (Chatzi and Smyth 2009) are a prominent representative of this class, offering the possibility for real-time identification of the damaged system parameters. This typically boils down to a joint state and parameter estimation problem, which may be solved by use of Bayesian filters, such as the extended Kalman filter, the unscented Kalman filter, sequential Monte Carlo (or particle filter) methods (Tatsis et al. 2022) or state-space observers such as the eigensystem realization algorithm and the observer/Kalman filter identification approach (Vicario et al. 2015). These methods are confronted with problems in the case of high-dimensional structures, i.e., systems of several degrees-of-freedom (DOFs). This issue may be alleviated via use of sub-structuring approaches for reduced order modelling. Bayesian filter approaches are particularly attractive for real-time implementation in the context of diagnostics and early warning systems.

## Residual life assessment (level 4)

Much research has been conducted in the area of damage identification, but linking damage to residual life prediction and decision support tools for the monitored structure is non-trivial, and remains challenging as the instances of permanent SHM deployments are still scarce.

One approach to quantifying the value of information stemming from monitoring data relies on use of Bayesian pre-posterior decision analysis. Omenzetter (2017) presents such a framework for detecting and classifying damage to infrastructure subjected to strong ground motion events for post-event decisions relating to safe operation and maintenance. This requires a priori modelling of the life cycle of an infrastructure that may be subjected to multiple seismic events. The infrastructure exposed to the main-shock or earlier aftershocks may suffer successive damage with resulting increase in vulnerability. SHM could be used to detect damage and accordingly inform decisions. This task is typically facilitated via use of decision trees for the visualization of root-cause analysis.

Indeed, perhaps the primary contribution of SHM in this respect lies in the evidence offered with respect to post-event condition. Upon updating of a relevant model (e.g., FE-based), seismic assessment for bridges may be achieved via computation of fragility functions. These define the probability of exceeding a specific damage state under a given earthquake intensity (Moschonas et al. 2009; Ramanathan 2012; Tecchio et al. 2016). In constructing these curves, different demand parameters in relation to a bridge component and typical damage types are defined (column deformation, abutment displacement, etc.). Nonlinear static or dynamic analyses are employed to derive such fragility functions, with a statistical analysis subsequently executed to define the exceedance of damage thresholds for each of the demand parameters. Fragility functions (Ozer et al. 2015) can be used for both pre-event and post-event decision making. As an actionable instance of the latter, the Shakecast system by Caltrans and USGS (Wald et al. 2008) couples vibration response data logged during and immediately after an earthquake with fragility functions to map the most likely impacted bridges from the specific event in the region of California.

Beyond assessment of events relating to extreme loads, monitoring may be used for calculating accumulation of deterioration due to fatigue or other slow evolving damage processes (e.g., corrosion). This is explored in the work of Chen and Wang (2013), who developed a probabilistic cumulative fatigue damage model for long-term monitoring of long-span suspension bridges under wind excitation. The method draws from using Bayesian learning and was validated on monitoring data from the Tsing Ma Bridge in Hong Kong. Alduse et al. (2015) also exploit a Bayesian scheme for assessment of the wind-induced fatigue damage of long-span bridges, while considering uncertainties related to available information on wind speed and direction. Remaining in the Bayesian context, Cho et al. (2017) propose a Bayesian correlation prediction model, relying on crack inspection data for analyzing the relativity of hydrogen-induced cracking in the cable wires of a steel suspension bridge.

## Computational intelligence in SHM

According to Engelbrecht (2007), computational intelligence is the branch of artificial intelligence dealing with “the study of adaptive mechanisms to enable or facilitate intelligent behavior in complex and changing environments". The interested reader can refer to (Russell and Norvig 2002) for a general overview on artificial intelligence. Techniques belonging to the area of computational intelligence have been largely employed for bridge monitoring applications in the last decades. In the following, we briefly review established applications of computational intelligence techniques for bridge structural monitoring applications (i.e., optimal sensor placement, damage detection, system identification, and model updating). Differently to the first sections of this work, which explained the essential levels of the SHM hierarchy, we devote this section to works dealing with evolutionary computing, swarm intelligence, and neural network architectures.

Evolutionary computation is grounded on the simulation of natural evolution processes, where the governing concept is the survival of the fittest individuals. On the other hand, swarm intelligence exploits the collective behavior of decentralized, self-organized natural systems of agents in problem solving. The swarm consists of a population of simple agents whose global behavior is not ruled by a centralized control structure that dictates how individual agents should behave. Conversely, the interactions among the agents lead to the emergence of an intelligent global behavior. Finally, neural network architectures, also referred to as neurocomputing, are based on a parallel and distributed information processing system that attempts to mimic how the human brain works.

Within the framework of bridge structural monitoring, evolutionary computing-based applications are mainly concerned with the use of the genetic algorithms (GA) (Holland 1992), while awarm intelligence- and neurocomputing-driven applications are linked to particle swarm optimization (PSO) (Kennedy and Eberhart 1995) and artificial neural network architectures (ANN) (McCulloch and Pitts 1943; Pitts and McCulloch 1947), respectively. Swarm intelligence includes applications of further, less popular algorithms, including the artificial bee colony (ABC) (Karaboga and Basturk 2007), the firefly algorithm (FA) (Yang 2009), and the monkey search algorithm (MA) (Mucherino and Seref 2007). On the other hand, most existing implementations of the ANN are based on the well-known feed-forward neural network (FNN). For a comprehensive, yet concise, review of GA, PSO and ANN (including their variants and relevant programming aspects), the interested reader can refer, for instance, to (Quaranta et al. 2020), where an extensive and up-to-date references list is also provided.

### Optimal sensor placement

The definition of the most appropriate sensor network layout is an important task when designing the structural monitoring campaigns. Formally, this is known as the optimal sensors placement (OSP) problem (Papadimitriou and Lombaert 2012). This consists in defining the optimal set of positions, among permissible locations, where a certain number of sensing units should be installed. Note that the type of sensors is typically defined in advance, whereas the number of sensors can be either an assigned design variable or can be itself an unknown. The design of the sensor network layout can thus be formulated as an optimization problem, in which the definition of the objective function plays an important role. Naturally, the sensors must be placed in such a way that the data they collect can provide adequate information about the structure (Limongelli 2003). As regards the analysis of bridges subjected to earthquakes, it means that the analysis of collected data from a sensor network must support as best as possible the tasks of identification, model updating and current condition assessment in view of the seismic reliability estimation, or the detection of damage that might occur after a seismic event. In several practical applications, it might be opportune to consider additional goals to define suitable sensor configurations. For instance, the OSP problem can also take into account direct and indirect monitoring costs, including development, purchase and maintenance costs for the sensors as well as resource and communication costs. Considering information gain in the search of good sensor configurations can also be useful to control over-instrumentation-related issues, i.e. when there is the need of taking into proper account the fact that too much information does not necessarily lead to significant benefits in terms of improved knowledge of the structure, since this can hinder a proper data interpretation. Moreover, robustness-based design criteria can be introduced to cope with potential malfunctions in the sensor network during its lifetime. Considering multiple goals in sensor network design might require accounting for conflicting optimum criteria. In such case, the OSP is most properly formulated as a multi-objective optimization problem, whose final solution will no longer be a unique set of optimal measurement points, but several alternative layouts that represent a compromise between the selected design criteria (so-called non-dominated optimal solutions). The OSP problem can be eventually completed by suitable constraints.

Although bridges generally comprise simpler geometries than more complex structures (such as large buildings), their dimensions and the significant contribution of many higher-order eigenmodes to their response typically demand a large number of sensors for structural identification and health monitoring. This renders task of defining an optimal sensor network layout challenging through use of empirically-based technical judgment or trial-and-error approaches. Owing to the growing complexity of the OSP problems to be solved in bridge monitoring, computational intelligence algorithms are increasingly employed to this end. We offer a short review of recent related works in this field. For a more extended discussion on the OSP problem and suited solution strategies, the interested reader can further refer to (Yi and Li 2012) and (Ostachowicz et al. 2019).

Rao and Anandakumar (2007) apply a hybrid swarm intelligence-based technique for the optimum positioning of sensors. The authors propose a modified version of the standard PSO algorithm, where the main novelty lies in introduction of a new user-defined control parameter from which the acceleration constants are determined. This parameter is then adjusted during the iterative search in such a way to privilege the exploitation of the best current solution or the exploration of the whole search space, depending on the distance between each particle and the best performer of the swarm. The PSO algorithm is also hybridized by applying periodically the Kelley’s variant of the Nelder-Mead algorithm (Kelley 1999) to the best 10% candidate solutions. A single-objective optimization problem is solved, with four alternative design criteria considered, namely maximization of the Fisher information matrix determinant, maximization of the strain energy matrix determinant, maximization of the kinetic energy matrix determinant, and minimization of the total mean square error of the mode shapes. The latter is estimated by comparing the mode shapes calculated from a FE model of the structure and those obtained through a cubic spline interpolation where a sensor is not positioned. The study concludes that network layouts optimized by means of the proposed computational intelligence-based approach are superior to those attained using classical deterministic procedures, with the possible exception of cases where the number of sensors is low.

You et al. (2013) address OSP design for a wireless sensor network for targeted to bridge SHM from two perspectives. On one hand, the optimal layout is intended to provide a reliable characterization of the dynamic behavior of the infrastructure and, to achieve this goal, the maximization of the Fisher information matrix determinant is considered. On the other hand, the design further aims at minimizing the total energy consumed by the wireless network. By resorting to the classical weighted sum method, these conflicting design criteria are combined into a single-objective function, whose optimal value is found through a modified PSO algorithm. Quaranta et al. (2014) compare GA and classical deterministic procedures for the design of the sensor network for a swing bridge. In this work, the optimal positions of the sensors are found by assuming in advance the number of measurement points. Hence, the MAC is employed to measure the difference between mode shapes estimated via FE analysis and those calculated by means of a cubic spline interpolation at the locations where a sensor is missing. So doing, network sizes corresponding to a value of the MAC less than 0.85 are discarded and the maximum number of sensors is selected among the remaining network sizes upon a cost-benefit evaluation.

Soman et al. (2014) address relevant practical issues in the design of complex sensor networks for bridge monitoring, that is the need of handling different types of sensors as well as the one of considering modal identification and damage detection tasks simultaneously. The ability of the network to ensure a reliable modal identification is quantified through the modal clarity index. The aptitude of the network in detecting existence and location of the damage is evaluated by using the mean relative error between the reference dynamics obtained from FE analysis and the one calculated after mode shape expansion via System Equivalent Reduction Expansion Process. These two goals are weighted to obtain a single objective function, and the final unconstrained optimization problem is solved using a discrete GA. The results carried out by Soman et al. (2014) suggest that, if properly optimized, the use of a network consisting of multiple types of sensors is beneficial. They also demonstrate that a global search strategy such as a GA can improve the overall quality of the sensor network layout compared to other conventional deterministic approaches.

The search for the optimal layout of a wireless network of sensors for bridge monitoring applications has been addressed in a series of articles by Zhou and co-workers. The strain energy is employed in (Zhou et al. 2014b, a) to define the objective function, and a constraint is included in such a way that the distance between two adjacent wireless accelerometers within a data transmission route is less than the assigned threshold transmission range. The resulting single-objective constrained optimization problem is then converted into an unconstrained one through a dynamic penalty approach. A GA and the FA are adopted to solve the resulting optimization problem in (Zhou et al. 2014b) and (Zhou et al. 2014a), respectively. The optimal placement of wireless sensors taking into account, both, the demands for a reliable bridge identification and the lifetime performance of the network was later considered also in (Zhou et al. 2015, 2017a). Such approaches are in line with the recent edge solutions Buckley et al. (2021a) that are being developed for monitoring.

A novel variant of the PSO and a chaotic MA have been implemented in (Li et al. 2015) and (Zhang et al. 2015a), respectively, to optimize the layout of a sensor network targeted at the modal identification of bridges. In both studies, the objective function aims to minimize the off-diagonal terms of the MAC matrix. Conversely, Zhou and Wu (2017) address the design of networks made of strain gauges essentially targeted at damage detection applications. In doing so, they introduce two indices to measure the capability of the strain gauges network in damage detection. One index corresponds to strain difference coverage; it directly takes into account the strain response changes due to a number of relevant damage scenarios, and aims at identifying the positions that maximize both damage coverage and detectability. The second index is the strain contribution coverage; it is based on the maximization of the Fisher information matrix determinant associated with the damage sensitivity matrix, which correlates strain response change and stiffness reduction under the assumption of a linear relationship between them. These indexes are then employed to select the best positions of the strain gauges from a discrete set of available alternatives by means of a GA. The results reported in Zhou and Wu (2017) highlight that the optimal layout is highly dependent on the load case (8 load cases are considered in this work, which are defined on the basis of truck numbers and moving paths). They also propose a means to derive a cost-effective number of strain gauges.

Recently, a modified version of the ABC algorithm is applied in (Yang and Peng 2018) aimed at determining the optimal location of sensors on bridges by minimizing the off-diagonal terms of the MAC matrix. The optimal placement of accelerometers and strain sensors is considered in (Wu et al. 2019), which is an extension of the previous work done by Zhou and Wu (2017). Notably, by selecting the strain gauge positions in order to optimize the strain difference coverage index for an existing bridge, the authors demonstrate that the damage detection capability of the resulting optimal network is much better than the one corresponding to the network of strain sensors already installed and designed on the basis of technical judgment only. As regards the optimal positioning of accelerometers, the final computer-aided design leads to monitor a larger number of in-plane DOFs of the deck with respect to the existing network already installed on the bridge. This seems counterintuitive, but the authors motivate such result by pointing out that the larger amplitude of the vertical deck response makes it more sensitive to changes due the damage. Hence, a smaller number of monitored DOFs is required in the vertical direction as compared to the in-plane direction. The authors also claim that too many sensors intended to monitor the vertical deck motion might be redundant due to the structural symmetry. Such a motivation, however, does not seem fully appropriate when the goal is damage detection since the faults, in general, can change the symmetry conditions.

### Computational intelligence for identification and model updating tasks

We previously referred to the value of model-based approaches for accurate prediction of the response and performance of bridge structures under seismic loads; a task which often involves the calibration/updating of structural models of existing bridges on the basis of experimental data collected from monitoring campaigns. In fact, large uncertainties might usually affect deteriorated and aging bridges, whereas reliable information about their boundary conditions are often unavailable. In view of this, computational intelligence methods have been successfully employed in (parametric or nonparametric) systems identification and model updating of bridges, with a short review of recent works presented in what follows. For a more extended review on the use of computational intelligence methods for system identification and model updating, the interested reader can refer to (Quaranta et al. 2020) and (Marwala 2010), respectively.

Huang and Loh (2001) apply a FNN trained by means of the Levenberg-Marquardt algorithm for the identification of a prestressed box-girder bridge under earthquake. The network is therein designed to produce a nonparametric model able to provide the seismic response of the infrastructure at the midspan of one girder and on the top of one pier, after a suitable training phase based on the experimental response available at nearby points. The correctness of the nonparametric modelling is then discussed through comparisons with available recordings. The authors conclude that the accuracy of the nonparametric model is not satisfactory for comprehensive damage detection, but that such a tool can be useful for recognizing unexpected changes in the structural response after an earthquake. Feng et al. (2004) also implement a FNN, which is differently purposed to what is presented by Huang and Loh (2001). The network designed by Feng et al. (2004) admits identified modal parameters as input, while the output consists in the structural parameters of the FE model of the bridge (such as the mass and stiffness elements). After extensive training and testing through FE analysis, the network is proven capable of identifying the structural parameter values based on the measured modal parameters.

Ataei et al. (2005) illustrate two further applications of the FNN. In the first reported application, a FNN is implemented to develop a nonparametric model able to predict bridge displacements under the passage of trains given measured strain time histories as input. The ability of the FNN to deal with such application has been demonstrated by comparing the experimental displacements with those obtained numerically by means of the trained network for train arrangements and velocity different from the ones considered in the training phase. Another application discussed in Ataei et al. (2005) comes up from the evidence that, if properly designed, the weights matrix of a linear ANN represents the flexibility matrix of the FE model at trained DOFs. This makes it possible to directly us on the use of ANNs for nonlinear identification and model updating of bridges are the weights matrix of the trained ANN for updating the FE model. A further confirmation of the ability of the FNN in identifying the dynamics of bridge structures from ambient vibrations can be found in (Chen 2005). Differently from these applications, the study by Li et al. (2010) is devoted to the identification, in a nonparametric way, of the relationship between modal parameters and environmental factors. In detail, the overall numerical procedure proposed in (Li et al. 2010) consists of two steps. First, a nonlinear PCA is performed to distinguish temperature and wind effects on structural modal parameters from other environmental factors. This enables the estimation of their contribution to the variation of the measured modal parameters, which are designated as output of a standard FNN. By assuming temperature and/or wind velocity as input of the FNN, nonlinear and nonparametric relationships between targeted environmental parameters and modal features are established.

Interesting contributions on the use of ANNs for nonlinear identification and model updating of bridges are reported in (Hasançebi and Dumlupınar 2013) and (Derkevorkian et al. 2014). Particularly, Hasançebi and Dumlupınar (2013) investigate the role of the nonlinearities on the reliability of the bridge model updating via standard FNN. In their work, the authors consider a small-span and deteriorated bridge for which a few experimental data were collected, namely three static displacement values and three natural frequencies. They then observe that the error rates obtained by means of a FE bridge model calibrated using nonlinear analyses data can be effectively reduced up to three times as compared when the model is calibrated using linear analyses data. Additionally, they highlight the fact that model updating is more accurate when both static and dynamic measurements are considered. On the other hand, Joseph and Pakrashi (2022) have recently demonstrated with simulations and experiments, how neuromorphic computing can be of particular relevance for detection of damage. Derkevorkian et al. (2014) also stress on the role of nonlinear response in developing nonparametric models for bridges. However, while Hasançebi and Dumlupınar (2013) have focused on the effects related to static displacements and natural frequencies measured under serviceability loading conditions, Derkevorkian et al. (2014) pay special attention on the energy dissipation assessment under seismic actions. To this end, a hybrid modelling approach is proposed in (Derkevorkian et al. 2014), where the equivalent linear part is modelled by means of a standard parametric least-square identification approach whereas the identified nonlinear forces are modelled using a nonparametric FNN-based methodology. The discussion in (Derkevorkian et al. 2014) supports the need of using nonlinear mathematical models for bridge identification to account for the contribution of energy dissipation sources other than the linear viscous one. Their results based on the experimental seismic response of a cable-stayed bridge demonstrate that nonlinear mathematical models are robust and stable when used for response prediction, while linear models are not sufficient for the estimation of the energy dissipation of complex structural systems like bridges, especially under high levels of dynamic excitation due to earthquakes.

Besides the use of ANN, GA and PSO are also common for system identification and model updating of bridges. In particular, the feasibility of GA for updating FE models based on the results of OMA is confirmed by many studies, see for instance (Lin et al. 2009; Ribeiro et al. 2012; Liu et al. 2016; Zhou et al. 2017b; Whelan et al. 2018). In all these studies, the objective function to be minimized through a GA aims at reducing the discrepancy between estimated and calculated natural frequencies and mode shapes simultaneously. Particularly, in the work done by Zhou et al. (2017b), a GA is also applied to minimize the difference between numerical and experimental data related to the static response of the bridge. Useful insights about the application of a GA for model updating based on static or dynamic tests are reported by Chisari et al. (2015), who consider a post-tensioned concrete bridge equipped with elastomeric bearing isolators. For both types of test, transversal and longitudinal stiffnesses of the isolators as well as pier and deck elastic modulus are updated by using a standard GA. As regards the model updating based on dynamic tests, the GA is employed to minimize two types of objective functions. In the first case, the objective function reflects the discrepancy in terms of bridge natural periods only. In the second case, bridge natural periods and mode shapes are taken into account simultaneously; the corresponding discrepancy measures are then considered altogether into a single aggregated objective function through the standard weighted sum approach, whereby different weights are employed in an attempt to estimate the whole Pareto front. By comparing the obtained results, the authors conclude that the discrepancy in terms of bridge natural periods only perform well in such case study, without significant loss of accuracy with respect to the case in which both natural periods and mode shapes are employed altogether for model updating. This result, however, is not very common and should not be considered to infer a general recommendation. A further outcome carried out by Chisari et al. (2015)—which maybe did not deserve the proper consideration—is the possible lack of convexity in certain regions of the final Pareto front estimated by taking into account bridge natural periods and mode shapes as concurrent model updating criteria via standard weighted sum approach. This seems to suggest that the weighted sum approach might be not the best technique for such application (while computational intelligence-based multi-objective optimizers would be more appropriate). Finally, Chisari et al. (2015) also point out that it is not possible to assess isolator stiffnesses by means of static tests only. Shabbir and Omenzetter (2015, 2016) also put together the discrepancy between estimated and calculated natural frequencies and that related to the mode shapes into a single objective function, but they also include a regularization term. By analyzing the sensitivity of a subset of relevant structural parameters for a cable-stayed footbridge, in fact, they observe that bearing stiffness and deck torsional stiffness have small influence on the experimentally identified mode shapes, with the only exception of the first torsional mode of the bridge. Without constraining these two model parameters, therefore, the problem can result ill-posed. They thus add a regularization (penalizing) term, in such a way as to keep the ratio between deck torsional stiffness and bearing stiffness almost constant during the model updating procedure. The final optimization problem governing the model updating is then solved by means of PSO (Shabbir and Omenzetter 2015) and GA (Shabbir and Omenzetter 2016). It is interesting to highlight that in all these studies, GA and PSO are applied to identify or update relevant macroscale parameters in bridge structures. In this sense, the work by Castro-Triguero et al. (2017) is somewhat different since it aims at calibrating the micromechanical parameters of a multi-scale model. In detail, Castro-Triguero et al. (2017) elaborate a multi-scale model for a timber footbridge and employ a GA to update the numerical values of degree of cellulose crystallinity, volume fraction of cellulose, volume fraction of hemicellulose, length of cellulose crystallites, radial dimension of wood cell, tangential dimension of wood cell, cell wall thickness, cell angle and microfibril angle. Throughout an iterative GA-based procedure, a homogenization technique is performed to obtain the macroscopic data for the global FE model of the bridge starting from these micromechanical parameters. The FE model, in turn, provides the estimated natural frequencies, which are compared with the experimental data during the iterative GA-based procedure to determine the optimal values of the micromechanical parameters. Further recent applications of ANN, GA and PSO in system identification and model updating of bridges from ambient vibrations have been presented by Qin et al. (2018); Sabamehr et al. (2018); Tran-Ngoc et al. (2018).

The state-of-art on use of computational intelligence techniques also includes successful applications pertaining to system identification and model updating of critical bridge elements, such as cables and hangers. For instance, Xie and Li (2014) address the identification of the tension force of bridge hangers. Specifically, a FE model of the hanger is developed, whereas its parameters (tension force, bending stiffness and boundary conditions) are identified simultaneously through a GA, which looks for the best match between numerical predictions of the hanger frequencies and the corresponding experimental values. The approach developed by Xie and Li (2014) is applied to hangers without and with dampers, by using laboratory test data as well as experimental data from field tests conducted on one hanger of a tied-arch bridge. The PSO is employed in (Dan et al. 2018) and (Xu et al. 2019a) for bridge cable systems identification.

### Health monitoring

The installation of a growing number of large sensor networks for continuous bridge monitoring has originated many studies towards the development of effective computational intelligence-based strategies for damage detection and health assessment (Sun et al. 2020). The availability of suitable sensing technologies at increasingly lower costs as well as the easy access to powerful computational resources has opened unprecedented opportunities in big data management for bridge health monitoring.

A multi-stage procedure has been implemented by Ko et al. (2002) to cope with the identification of damage occurrence, localization, and magnitude. The proposed novelty detection technique adopts an ANN architecture, in a first stage, to deliver alarms on the possible occurrence of damage. The framework operates on the basis of tracking the natural frequencies of the structure. In a second stage, damaged members are identified on the basis of normalized index vectors derived via exploitation of the well-established concepts of modal curvature and modal flexibility. In a third stage, an ANN is used to detect damaged member(s) and to further quantify the extent of damage. In a similar context, Lee et al. (2005) feed the differences or the ratios of the mode shapes evaluated prior to and post damage to an ANN-based architecture; these features are considered less sensitive to modelling errors than the mode shapes themselves. The designed ANN outputs the element stiffness ratios prior to and after damage. Koh and Dyke (2007) adopt a GA scheme in combination with a multiple damage localizing assurance criterion in order to locate the damage on a real cable-stayed bridge structure. The robustness of the approach is verified through numerical simulations. The results further reveal the challenges stemming from availability of only a few mode shapes in quantifying the damage extent in complex infrastructures comprising multiple potentially damaged structural elements. Li et al. (2008) apply several ANNs with different inputs to gain an initial estimation of the damage. Then, optimal weighting coefficients obtained by means of a GA are assigned to the ANNs. In this context, the Dempster-Shafer evidence theory and the Shannon entropy are employed for information fusion and to reduce the impact of uncertainties on damage identification, respectively.

Perera and Ruiz (2008) put forth a framework that is specifically aimed at bridge health monitoring, which draws from the work of (Ko et al. 2002), in that it employs a multi-stage procedure for damage detection, localization and quantification. A main driver of this work lies in the adoption of a multi-criteria context for damage identification, with modal flexibilities and a damage localization criterion, relying on modal properties, considered as conflicting objective functions. Park et al. (2009) address the problem of identifying the distribution of stiffness reduction in damaged bridges under moving loads. A modified bivariate Gaussian distribution function is herein proposed to simulate the stiffness reduction due to cracks, whose parameters are identified through a GA that employs a reduced population size (i.e., a micro-GA). On the other hand, further applications of the ANNs for health monitoring of bridges are presented in (Bagchi et al. 2010) and (Min et al. 2012).

The need for removing the disturbance due to environmental factors in order to reduce false positives and false negatives in bridge damage detection is tackled in (Zhou et al. 2011) and (Meruane and Heylen 2012). Specifically, Zhou et al. (2011) employ two different ANNs to cope with the removal of environmental effects and damage detection. Like (Ko et al. 2002), Zhou et al. (2011) adopt a special kind of ANN for damage detection, namely an autoassociative ANN. Relevant features from the monitored structure in healthy conditions (e.g., modal features of the healthy structure normalized with respect to changing environmental conditions) are used during the training stage. Once the training process is completed, the same set of structural features is passed again to the autoassociative ANN in order to generate its reproduction, and the reconstruction error is evaluated. Once a new set of structural features pertaining to an unknown state of the structure is identified, it is fed into the trained autoassociative ANN to yield its prediction and, consequently, the reconstruction error. If the structure is undamaged, then no significant variations in the reconstruction error will be observed, whereas the opposite will imply the possible occurrence of damage if the variation is larger than a suitably selected threshold.

Mosquera et al. (2012) study the feasibility of exploitation of strong motion data recorded on bridges subjected to earthquakes and aftershocks toward assessing structural integrity. In their study, an eigensystem realization algorithm with an observer Kalman filter is used to identify the modal parameters of the bridge from low level earthquake excitation. Next, a FE model of the bridge is updated. To this end, a GA is tasked with updating selected model parameters so as to minimize the difference between simulated and experimental values of the natural frequencies. The updated FE model, in turn, is employed within a pushover analysis to estimate the displacement values at different performance levels, which are then compared against the measured displacements, under strong earthquake events, in order to assess potentially damaged areas. Wang et al. (2013) develop a multi-layer GA for damage detection in large truss bridges. In this strategy, the elements of the truss structure are first categorized into subgroups depending on their position (e.g., upper chord, lower chord, vertical webs, diagonal webs). The algorithm adopts the modal strain energy correlation (MSEC) as the objective function and seeks to identify damaged elements within the subgroups, with the converged population in each group serving as the initial population of a subsequent layer, where the groups are redefined through merger of the previous smaller subgroups. From a computational standpoint, the authors claim that this approach offers advantages over the standard GA, improving convergence and reducing the probability of entrapment into local optima due to the restart of the evolutionary search in each layer of the GA scheme.

A bridge damage detection procedure based on ANN that exploits statistical parameters of train-induced vibrations as input has been proposed by Shu et al. (2013). This is motivated by the fact that changes in the modal features at the onset of damage are hard to identify in complex structures due to their inherent redundancy. Additionally, the authors point out that the use of the measured bridge dynamic response does not require a preliminary processing of the time records to identify modal features, thereby simplifying the whole health monitoring procedure. Based on these premises, Shu et al. (2013) initially prepared a training set by using a simplified FE model and considering different stiffness reduction levels. Throughout extensive numerical investigations, they thus evaluated the reliability of the damage detection procedure regarding damage location, noise level, train type and speed. As expected, the results confirm that the accuracy of the damage detection procedure enhances significantly at resonance loading speeds, mainly because this hides the disturbance due to the noise. Results also demonstrate that this approach can detect a minimum element stiffness reduction of about 10%. Other pure data-driven bridge damage detection approaches based on ANNs are proposed, for instance, in (Neves et al. 2017) and (Weinstein et al. 2018).

Two recent applications of the GA in bridge damage detection are reported in (Conde et al. 2016) and (Silva et al. 2016). These works, however, make use of the GA in a different way. The application in (Conde et al. 2016) follows previous studies in which the GA is adopted to adjust the parameters of the mechanical model of the structure so that an error measure based on the differences between real and numerically predicted damage pattern is minimized. Conversely, the use of GA by Silva et al. (2016) roots on the idea that damage detection problems can be formulated as clustering problem. On this basis, they advance a GA-based approach for damage detection in bridge structures that consists of two main steps. First, the normal state conditions are discovered automatically by clustering a training dataset according to the closest centroids, which are targets of the optimization task performed using GA in a way that defines boundary regions between the clusters and reduces the number of discovered state conditions. At a second stage, the damage detection strategy takes place by exploiting the Euclidean distances between the new observations and the optimized centroids (the minimum distance to the centroids thus represents the damage indicator).

More recent advances and novel applications of the ANN in bridge health monitoring are reported in (Tran-Ngoc et al. 2019), (Neves et al. 2021) and (Rageh et al. 2020). Particularly, Tran-Ngoc et al. (2019) investigate the role of the frequency content in ANN-based damage detection of railway bridges under serviceability loads. Low- and high-frequency contents of recorded signals (e.g., accelerations) are attributed to global structural dynamics (from which modal features are thus derived) and train-track-bridge interaction phenomena (mostly governed by track irregularity), respectively. In their study, Neves et al. (2021) recommend putting more attention on the possibility of using high-frequency content for damage detection on bridges. The work by Rageh et al. (2020) is especially worthy of mentioning because it is one of the few existing studies about fatigue-induced damage detection in steel bridges.

Compared to ANN and GA, there are less investigations about the use of swarm intelligence techniques, such as in (Li et al. 2017), (Huang et al. 2019) and (Cancelli et al. 2020). In detail, Li et al. (2017) illustrate the application of the acoustic emission technique to assess corrosion-induced damage in steel wires used in bridge cables and employ a PSO-based clustering algorithm to infer which kind of corrosion has occurred. Huang et al. (2019) formulate the bridge health monitoring as an optimization problem in which the PSO algorithm is adopted to look for a minimum of an objective function that combines natural frequencies, MAC and modal strain energies as damage sensitive features. This strategy has been tested considering the effect of temperature variations, but there are neither effective guidelines to weight the different damage features into a single objective function nor sensitivity analyses that demonstrate how the final solution depends on the numerical values of the weights. A similar approach was basically presented in (Cancelli et al. 2020), but different objective function formulations to be minimized via PSO are considered depending on the targeted damage sensitive feature.

Among the most recent applications of neurocomputing techniques in bridge health monitoring, autoencoders are swiftly gaining in popularity. The autoencoders (also known as auto-associative ANNs) are deep neural nets (i.e., ANNs with more than three layers in total) that include a dimensionality reduction operation, termed the encoder (which reduces the dimensionality of the input data fed to the network to a latent space), and a reconstruction operation, termed the decoder (which aims at reconstructing in the output layer the originally fed input). In this context, Lee et al. (2019) propose an autoencoder-based framework to detect tendon damage in prestressed concrete bridges. In this and similar works (Sarwar and Cantero 2021), the autoencoder is basically employed as a feature extractor, or novelty detector. Briefly, the autoencoder is first trained using data collected from the undamaged structure, and the reconstruction error (i.e., a measure of the difference between the output and the input of the autoencoder) is estimated. Next, the trained autoencoder is applied to current data: a significant variation of the reconstruction error from the settled reference range is attributable to a variation of the structural properties, such as damage. Lee et al. (2019) consider simulated acceleration data for a prestressed concrete bridge under traffic load, and conclude that the best result is obtained under the passage of a single vehicle while large damage levels only can be identified accurately in case of multiple vehicles crossing the bridge. An experimental validation has been later presented by Lee et al. (2021) using both acceleration and strain data. The extraction of damage-sensitive features via autoencoders for damage detection in bridges is also addressed in some recent works (Silva et al. 2021; Shang et al. 2021).

Another class of neurocomputing techniques that is rapidly attracting consideration are the generative adversarial networks. They consist, on the one hand, of a first ANN (generator) that produces new samples from random noise. On the other hand, a second ANN (discriminator) discerns between real and fake data. After a successful training stage, the first neural model is expected to generate samples that appear very similar to the real ones. The underlying mechanism is thus a kind of two-people zero-sum game where, at the end of the training phase, the Nash trade-off equilibrium is achieved (i.e., the total gains are zero for both the players, and loss or gain of the utility for each player stalls to a balanced level). In the field of bridge monitoring, generative adversarial networks are mainly employed for data augmentation or reconstruction. For instance, Luleci et al. (2022) implements generative adversarial networks for generating synthetic labelled acceleration data in the aim to overcome data scarcity in vibration-based bridge damage detection. Lei et al. (2021) and Gao et al. (2022) adopted generative adversarial networks to reconstruct lost strain and displacement data for SHM of bridges in case of transmission failure or sensor fault. The combination of generative adversarial networks and autoencoders toward data anomaly detection for automated SHM of bridges has been discussed by Mao et al. (2021). For a general review about the generative adversarial networks in earthquake engineering at large, the interested reader can refer to (Marano et al. 2023).

### Vision-based SHM

We separately here refer to the use of vision-based SHM schemes, as an emerging non-contact assessment approach (O’Byrne et al. 2015), which particularly benefits from use of computational intelligence methods. These have also been extend to colour O’Byrne et al. (2014, 2018) and texture based methods O’Byrne et al. (2013), with further application to bridges O’Donnell et al. (2017), creation of benchmark repositories O’Byrne et al. (2018c), use of virtual imagery O’Byrne et al. (2018b, 2020) and underwater detection O’Byrne et al. (2018a). This approach has in recent years gained ground for assessment of structural damage, especially for bridge structures, where relevant data can now be made available via use of unmanned aerial vehicles (Aldana-Rodríguez et al. 2021; Valença et al. 2017). Several works that relate to vision-based monitoring data focus on the task of crack identification, which may be achieved via machine-learning based image processing and segmentation tools (Quqa et al. 2022; Xu et al. 2019b). Such information can be further fused with the damage criteria, overviewed in earlier sections of this paper, in order to infer damage. A criterion that is specific to displacement information, relates to influence lines, as discussed in the work of Liang et al. (2017). On the other hand, vision-based information can also be linked to modal information (such as modal frequencies and mode shapes) through so-called magnification tools, which then permit the coupling with damage detection criteria developed for vibration-based SHM. Finally, the fusion of vision-based information with conventional sensing systems, can serve as a booster for the identification task. In this direction, Zaurin and Necati Catbas (2011) and Zaurin et al. (2016) experimentally pointed out the value of fusing video or image data with conventional monitoring data (e.g., accelerometers) for condition assessment of bridge structures; a task that is particularly valuable for post-earthquake damage assessment.

## Practical instrumentation aspects of long-span suspension bridges

In the previous sections, a general review of existing methodologies was presented regarding the structural health assessment of instrumented structures through the recordings of their dynamic response. In the following, some practical instrumentation issues are also presented, through a survey of the SHM systems actually implemented (and planned to be further updated) on two long-span suspension bridges in Istanbul, at the high seismic hazard Marmara Region, Turkey. Furthermore, SHM data acquired at one of the bridges after a low intensity seismic event (the $M_w$ 5.8 Silivri Earthquake of September 26th, 2019) are used to evaluate its dynamic characteristics, and to assess the possibility of damage due to the earthquake. The analyses carried out in the present case are to be considered as basic examples aiming to point out some general concepts of the assessment procedures. For more in-depth analyses, several of the more advanced methodologies presented in the previous sections can be implemented here as well as in other actual situations with different levels of seismic intensity, structural complexity and data availability. From such incidents, it becomes evident that a bridge’s structural integrity can be rapidly assessed after a seismic event without need for disrupting its operation. The overviewed instrumentation cases exemplify the role of SHM systems in ensuring the functionality and safety of bridges and other critical infrastructures.

### SHM systems for long-span bridges

A significant number of studies on deployment of SHM systems for long-span bridges have been reported globally (e.g., Aktan et al. 2002; Brownjohn 2007; Habel 2009; Ko and Ni 2005; Mufti 2002; Wenzel 2008; Wong 2007; Xu and Xia 2011; Xu et al. 2000; Chen et al. 2004). Wong (2007) proposed a rational approach for designing SHM systems for long-span bridges. Connections between SHM systems and relevant issues such as bridge management, maintenance, and life-cycle performance assessment have been widely studied as well (e.g., Catbas et al. 2008; Wong and Ni 2009; Frangopol 2011; Fujino and Siringoringo 2011; Frangopol et al. 2012; Wang et al. 2016).

The wide diffusion of continuous SHM systems for long-span bridges can be attributed to the importance of such infrastructures within the transportation network they belong to, which can eventually extend beyond the national borders. In this sense, Istanbul’s metropolitan area provides a truly emblematic case: due to its strategic location straddling the Bosphorus Strait, long-span bridges in this area provide a critical link between Asia and Europe from which the mobility needs of persons and goods depend heavily on. Due to the seismic hazard in the Marmara region that may create $M_w > 7$ earthquakes and the proximity of the most active fault zone in Turkey, the North Anatolian Fault Zone (NAFZ), to the inventory of long-span bridges in the region, periodic maintenance and health monitoring of these bridges are essential to mitigate seismic risk. Further details regarding bridges’ SHM systems and the local seismicity can be found in (Memisoglu Apaydin et al. 2022). A summary of the description, instrumentation and the existing experimental investigations for the First and Second Bosphorus Bridges based on SHM systems is provided along with the design considerations in the following.

### Design considerations for the SHM systems

Strong earthquakes, winds or typhoons, extreme traffic conditions (i.e., heavy truck loading), marathons (i.e., human-induced loading), and extreme thermal loading were considered relevant by the Turkish State Highways General Directorate (KGM) when designing the SHM systems for long-span bridges. Hanger load variation, deck expansion, bridge response (i.e., the response of tower, deck, cable, and hanger), deck fatigue (due to traffic), environmental input (wind and thermal loading), and general geometry are the aspects that were recognized as relevant for the SHM systems design. Rapid issuing of reports on the bridges’ conditions after extreme events that provide information about their operational state in terms of fatigue, deformations, and stresses as well as about their geometry and performance in case of future extreme events is the main goal pursued by KGM when setting up the SHM systems. Additionally, the SHM system was required to track critical response parameters to determine whether they exceeded a threshold value corresponding to a certain design limit. The threshold is set for each component of the bridge. When the threshold is exceeded, an alert is issued. Then, SHM data are collected and processed, and a preliminary report is elaborated for the KGM. General objectives and recommendations for the SHM systems were presented by Aktan et al. (2002). Among others, the SHM system’s durability was deemed a critical issue and required special design considerations that also included a periodic preventive maintenance program.

### Description and instrumentation of the first Bosphorus bridge

**Fig. 2:**
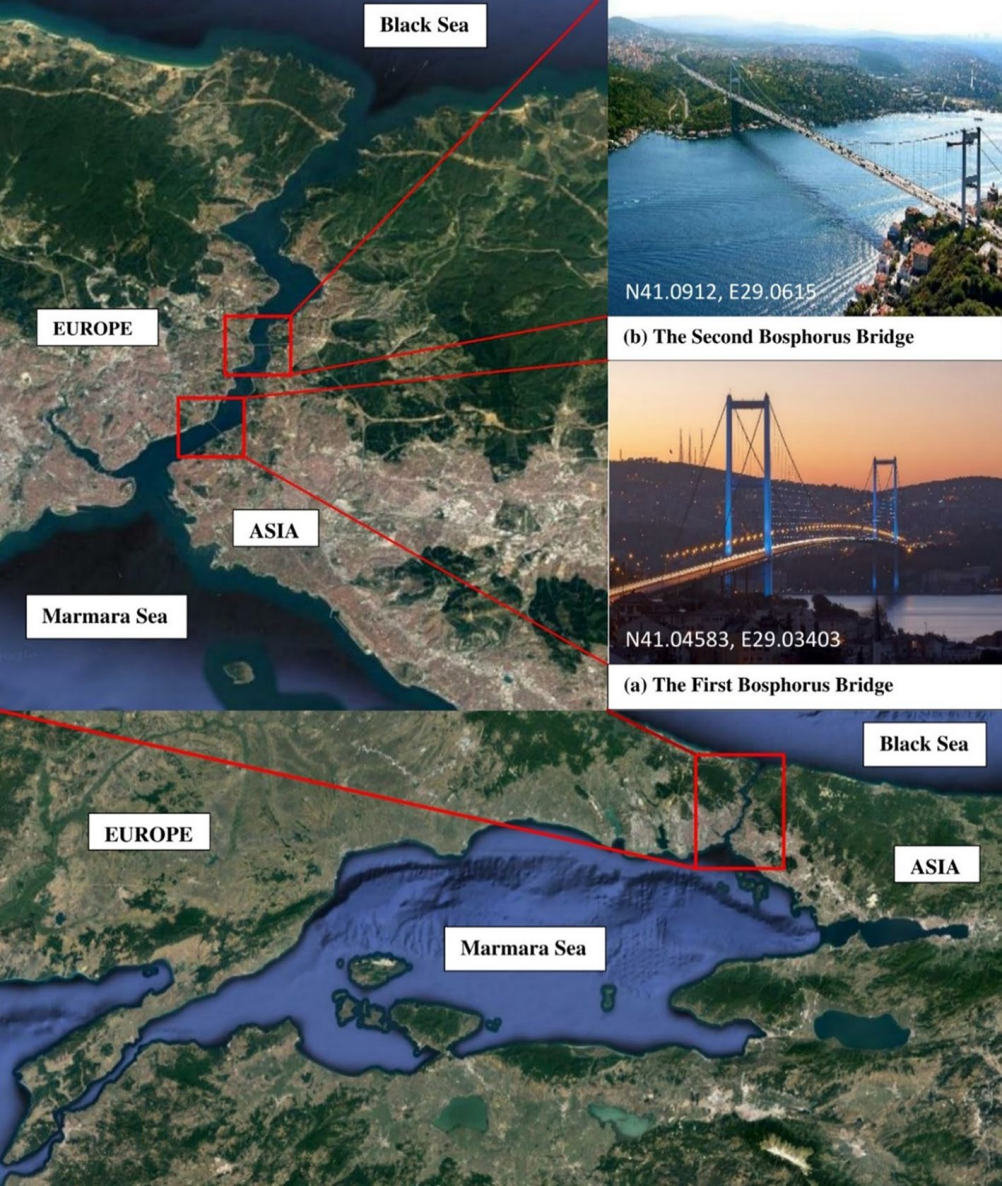
A general overview and location of long-span bridges: **a** the First Bosphorus Bridge (the 15 July Martyrs Bridge), **b** the Second Bosphorus Bridge (the Fatih Sultan Mehmet Bridge)

The idea of constructing a bridge over the Bosphorus Strait is centuries old. However, the first decision was made in 1957. The structural model was developed in 1968 by Freeman Fox & Partners. The bridge was opened on the 50th anniversary of the Turkish Republic, which is about three years after its construction began in 1970. When the construction was completed in 1973, it was the fourth longest bridge in the world. The bridge is now the 40th longest suspension bridge in the world and is still an important part of Istanbul’s transportation network. A general overview of the First and Second Bosphorus Bridges and their position in the Marmara region are shown in Fig. 2.

**Fig. 3:**
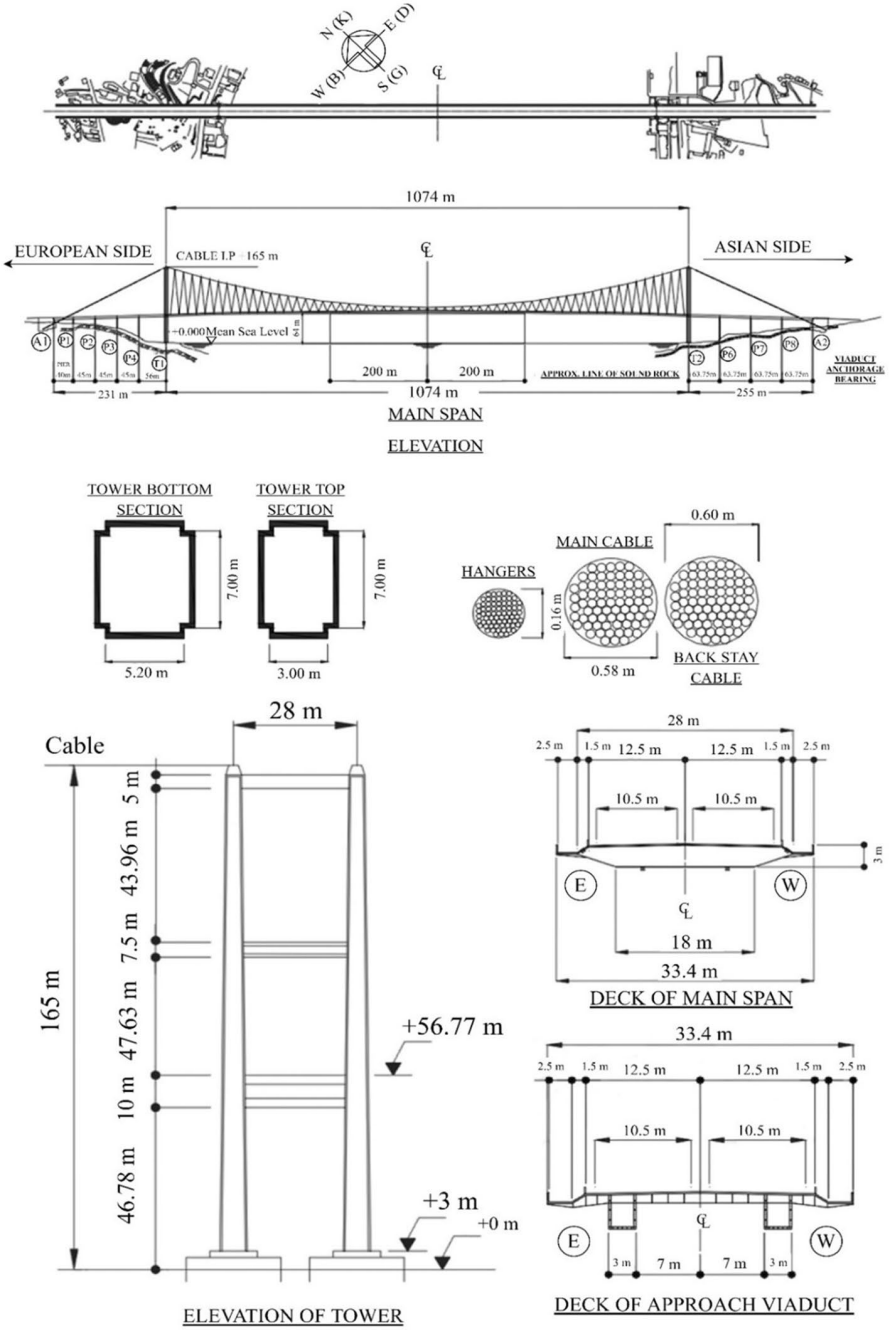
General layout and dimensions of the First Bosphorus Bridge

The First Bosphorus Bridge is an eight-lane steel suspension bridge with three regular, one emergency, and one pedestrian lane in each direction. The bridge is a gravity-anchored long-span suspension bridge with steel towers and an aerodynamic box section deck (Fig. 3). The aerodynamic box section maintains the deck, reduces the wind impact by 1/3, and requires less material than the traditional truss system. The length of the main span is 1,074 m, and there are two approach viaducts, the Ortaköy viaduct on the European side and the Beylerbeyi viaduct on the Asian side.

These approaches have five and four spans with a length equal to 231 m and 255 m, respectively. The main span of the bridge is supported by hangers, whilst the viaducts are supported at the base by columns of varying heights. Additionally, they are simply supported on the ground. The deck has a width of 33.40 m, whereas the height is about 3 m. As it can be seen in Fig. 3, the towers are 165 ms high from sea level to the saddle. The equivalent diameter of the hanger cable with an angled pattern is 0.06 m, whereas the main and backstay cables have a diameter roughly equal to 0.58 and 0.60 m, respectively. Additionally, the approach span of the bridge has a rigid superstructure consisting of a continuous rectangular box section supported by cross beams and a circular box of columns. The bridge operation remains vital nowadays, given its yearly traffic volume of more than five billion vehicles. Due to the fact that commercial vehicles such as trucks are routed through the Fatih Sultan Mehmet (FSM) bridge, the First Bosphorus Bridge is only opened to cars and buses. Additionally, pedestrians are no longer permitted to use the bridge, as they were for the first three years after it was opened to vehicles. Exceptionally, the annual Intercontinental Istanbul Eurasia Marathon takes place in October, and the bridge, closed to traffic during the event, receives an overwhelming foot loading.

A temporary SHM system was considered in some initial researches (Petrovski et al. 1974; Tezcan et al. 1975; Erdik and Uçkan 1989; Brownjohn et al. 1989), where the significance of a continuous SHM system was also highlighted. Therefore, the first permanent SHM system was installed in 1993, and it was later upgraded to ensure smooth operations. The SHM system comprises accelerometers, tiltmeters, force transducers, strain gauges, laser displacement, global positioning system GPS, thermocouples, and weather stations. A total of 168 sensors and 258 channels were positioned strategically on the bridge, considering its general features (Bas et al. 2018). Using sensors and real-time data acquisition modules, the SHM system captures essential information about the bridge response in daily and extraordinary circumstances. The types of sensors, their main specifications, and their quantities are listed in Table 1.

**Table 1:** Sensor types, specifications, and number for the SHM system of the First Bosphorus Bridge

Sensor type	Specifications	No. of units
Force transducer	Measurement range: $\pm 1.50$ mm Repeatability: $0.30 \times 10^{-3}$ mm/m Linearity: $0.30 \times 10^{-3}$ mm/m Operating temperature: $-10$ to 80 ^∘^C	12
Accelerometer	Measurement range: $\pm 2.00$ g Sensitivity: 2000 mV/g $-3$ dB frequency cutoff: 300 Hz Shock survival: 2000 g	19
Tiltmeter	Measurement range: $\pm 14.50^\circ$ Resolution: $1^{\prime \prime }$ $-3$ dB frequency cutoff: 5 Hz Shock survival: 1000 g	15
Strain gauge	Resistance tolerance: $\pm 0.30$% Gauge factor: $\approx$2 Operating temperature (static): $-70$ to 200 ^∘^C Operating temperature (dynamic): $-200$ to 200 ^∘^C	70
Weather station	Wind spped range: 0–130 mph Threshold sensitivity: 2.40 mph Pitch: 29.4 cm air passage/revolution Operating temperature: $-50$ to 50 ^∘^C	6
Laser displacement	Measurement range: 200-2000 mm Resolution: 1–3 mm Max measurable frequency: 10 Hz Operating temperature: $-20$ to 70 ^∘^C	8
Thermocouple	Thermocouple type: J Accuracy: $\pm 0.10$% Support type: fixed on copper or steel collar	33
GPS	Precision: 0.2 mm Operating humidity: up to 95% Sampling rate: 10 Hz Operating temperature: $-40$ to 65 ^∘^C	5

**Fig. 4:**
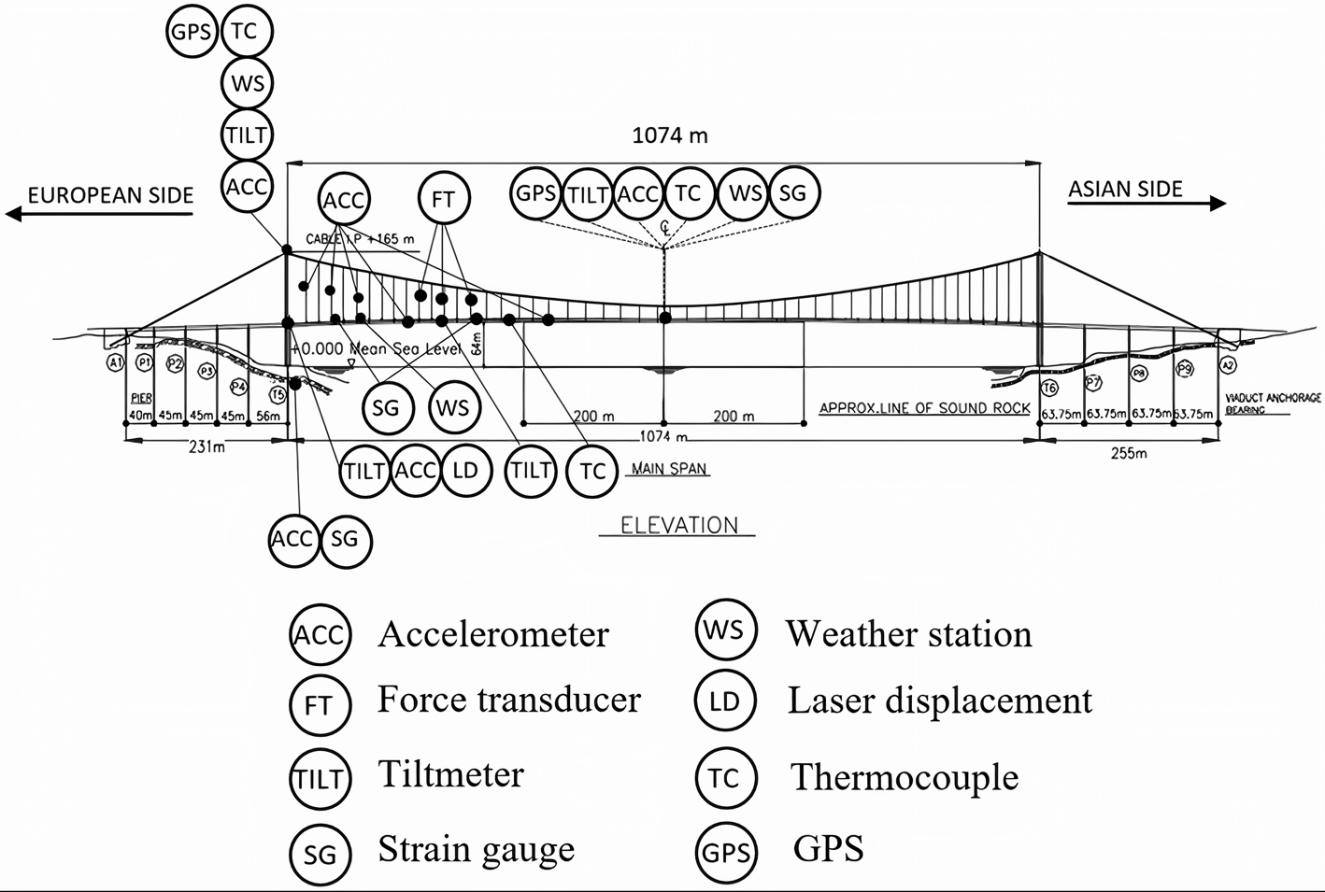
Sensors arrangement for the SHM system of the First Bosphorus Bridge

The sensors’ position is illustrated schematically in Fig. 4. It is noted that the First Bosphorus Bridge had inclined hanger elements instead of a typical vertical hanger arrangement until 2015. Hanger elements and stool plates are equipped with 14 strain gauges and 12 force transducers, together with 6 accelerometers for vibration monitoring (KGM 2008a, b). Bas et al. (2018) provide further information about data acquisition components such as site supervisor software, backup computers, and data acquisition hardware.

### A brief survey on the first Bosphorus bridge

The earliest experimental studies on the First Bosphorus Bridge were carried out once it was opened to service in 1973. These initial studies mainly focused on identifying the modal characteristics of the bridge from ambient excitation and forced vibration tests (Petrovski et al. 1974; Tezcan et al. 1975). A limited number of sensors were employed in those experimental studies. Three accelerometers were installed on the bridge to monitor its response under wind load, and a forced vibration test was performed utilizing two shakers mounted in the middle and at the quarter of the bridge span. Another experimental investigation was carried out later by Erdik and Uçkan (1989), and its results confirmed the previous studies’ findings. A comprehensive experimental study of the First Bosphorus Bridge was performed by Brownjohn et al. (1989). Experimental and numerical results were compared, and a reasonable agreement was obtained in terms of the spectral density and transfer function under wind and traffic loads. The first permanent SHM system for the First Bosphorus Bridge was installed in 1993 and included three different subsystems, namely sensors, a data acquisition system, and data recording-monitoring systems. The sensor subsystem included accelerometers, seismometers, wind speed, and direction meters for a total of 28 channels. Another ambient vibration test was conducted by Beyen et al. (1994) in order to compare new experimental data with existing results, and a satisfactory agreement was found.

More recent works were conducted starting in the 2000 s by Apaydin and Erdik (2001); Erdik and Apaydın (2007); Kosar (2003). Bas (2017) analysed SHM data collected during a strong wind event. The First Bosphorus Bridge’s model was validated considering the difference between the experimental results and the numerical predictions obtained from a FE model. Apaydın (2010) provided free vibration analysis for the First Bosphorus Bridge. SHM data acquired during a storm on 18th April 2012 were considered by Apaydin et al. (2013). Particularly, they compared the experimental results with those collected under usual weather conditions. This analysis revealed that the bridge’s natural first period increased during the considered extreme wind event. Based on the results of NDE tests, after the extreme wind event in 2012, KGM decided on a hanger replacement in 2015 due to insufficient bearing capacity. Bas et al. (2020) determined the hanger element replacement effect on the First Bosphorus Bridge’s structural response under a multi-support seismic ground motion. The FE model they adopted was validated using SHM data recorded earlier during the extreme wind event dating back to 2012. The First Bosphorus Bridge model identification during hanger replacement by means of ambient vibration data was performed by Soyöz et al. (2020).

### Description and instrumentation of the Second Bosphorus Bridge

**Fig. 5:**
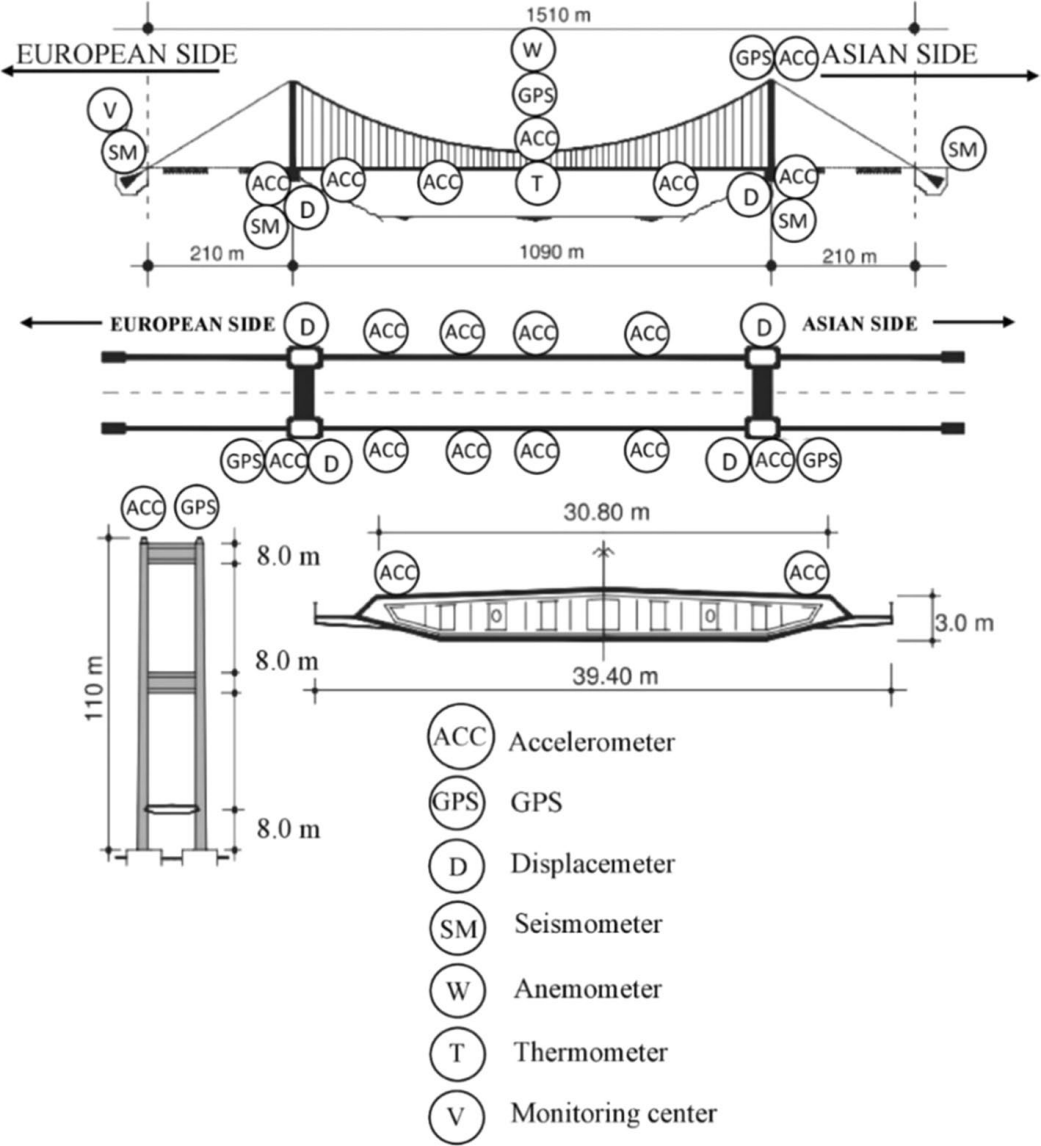
General layout, dimensions, and the SHM system of the FSM Bridge

A second long-span bridge across the Bosphorus Strait became a necessity after the decision to build the Trans-European Motorway (TEM) in Istanbul. Thus, the British firm Freeman Fox & Partners was assigned to prepare for the second bridge project. The construction was completed in 1988, and it was the 5th longest suspension bridge in the world at that time. It is now ranked as the 36th longest suspension bridge. The Second Bosphorus Bridge is a steel long-span suspension bridge located on the north side of the First Bosphorus Bridge. The towers are supported at the ground level. Since the end of the deck is at the level of the tower base, the bridge was designed with no approach spans. The main span is 1090 m long, whereas the deck is 39.40 m wide and 3.00 m high. The tower is 110 m high and has a rectangular box section. The mid-span deck is 64 m above the mean sea level. The bridge is 1510 m long, including side spans, each having a length of 210 m. The bridge is susceptible to substantial traffic loads since the TEM highway is the only route available for trucks and other large vehicles. A permanent SHM system was first installed on the FSM Bridge in 2001. Figure 5 shows the general layout, dimensions, and the SHM system’s sensor position on the FSM Bridge.

The SHM system consists of 12 accelerometers, 4 displacement meters, 2 seismometers, 2 GPS, 1 weather station, and 1 thermometer (Table 2). The data collection system SEISLOG is used, and two parallel PC systems monitor the data acquired from 64 channels with a resolution equal to 12–24 bits. Besides, the SHM system has two different AC/DC converter boards (KGM 2001).

**Table 2:** Sensor types, specifications, and number for the SHM system of the FSM Bridge

Sensor type	Specifications	No. of units
Accelerometer	Measurement range: $\pm 0.59$ g Frequency range: 0–50 Hz Filter: 50 Hz Butterworth filter	12
Seismometer	Frequency range: 0.5-$-$1.0 Hz Components: lateral, vertical	2
Displacement meter	Capacity: 50 cm Resolution: 0.01 cm	4
GPS	Position update: up to 10/s Position latency: 30 ms	2
Data acquisition	A/D converter: 12–24 bit Sampling rate: 200 kHz	1

**Fig. 6:**
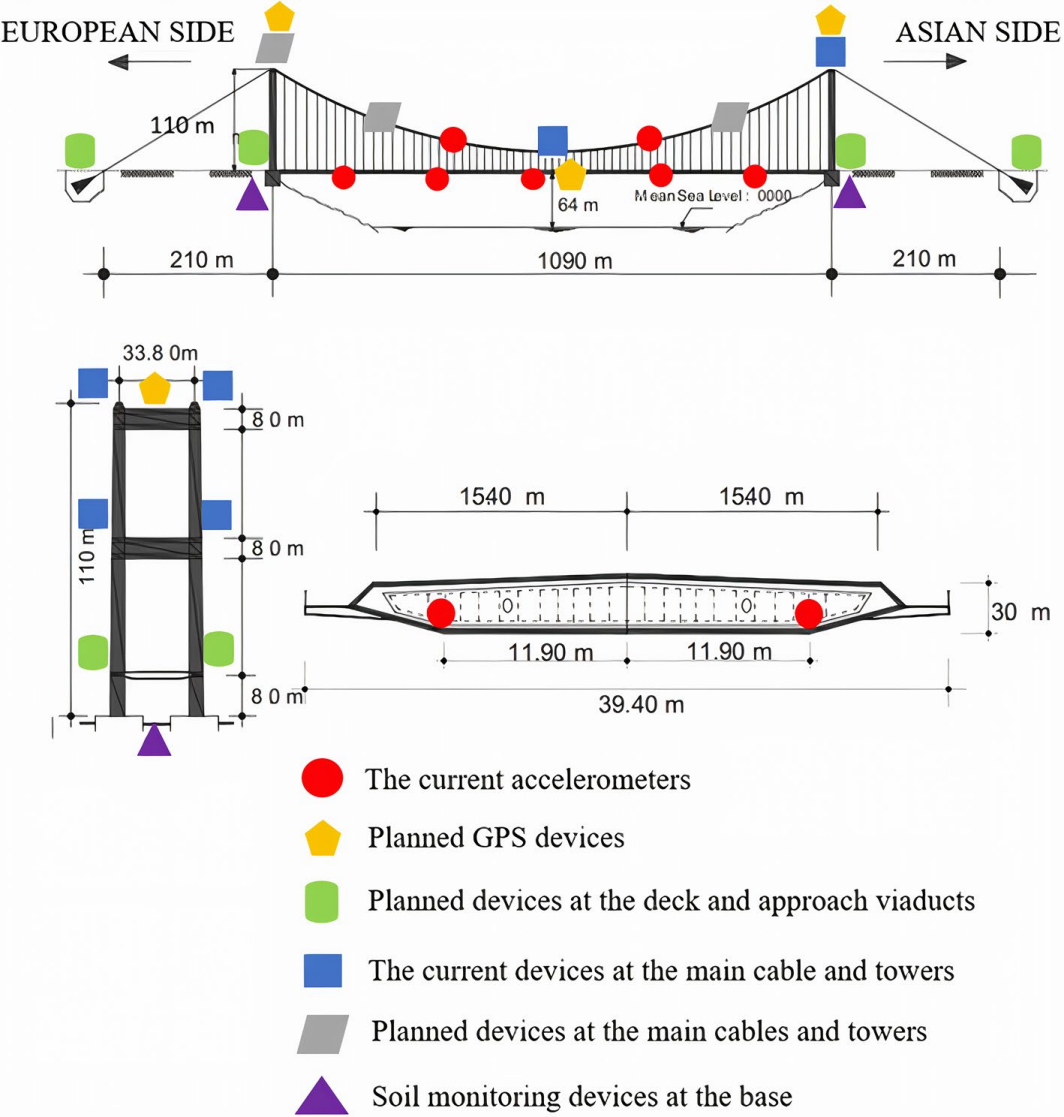
The new planned SHM system for the FSM Bridge

Due to the FSM Bridge’ strategic position along the TEM, such an infrastructure is subjected to heavy truck loading. Even though it is only allowed during a certain time period in a day, the traffic load is heavier than on the First Bosphorus Bridge. Since the design specifications require high expansion joint movement capacity, all displacement sensors on the bridge are deployed at the expansion joints. At the ground level, four seismometers are mounted on the support points of back-stay anchorages and tower legs. KGM conducted a preliminary study aiming to improve the permanently installed SHM system for the FSM Bridge. The schematic configuration of the new planned SHM system is shown in Fig. 6. Notably, cable elements, expansion joints, and anchorage points are all equipped with new sensors.

### A brief survey on the Second Bosphorus Bridge

The Second Bosphorus Bridge has been subjected to experimental studies since the 1990 s. The first dynamic monitoring applications for this bridge are reported in (Brownjohn et al. 1991, 1992a, b). These studies aimed to identify the modal characteristics in terms of natural frequencies, mode shapes, and modal damping ratios using data collected from decks and towers under wind and traffic loads. Although lateral modes were not detected due to the low response of the bridge, vertical and torsional modes were identified accurately. In addition, a comparison between the experimental results and those from a two-dimensional FE numerical model was also presented. The FSM bridge was later equipped with a new SHM system by installing 32 triaxial accelerometers at the critical points of the bridge (Apaydin and Erdik 2001). Seismometers, displacement meters, GPS sensors, anemometers, and thermometers were also installed to monitor wind, traffic, earthquake, and thermal effects on the bridge. A three-dimensional FE model of the bridge was also developed by Apaydin (2002). Vertical, lateral, and torsional vibrational modes and their corresponding frequencies were extracted from SHM data and compared with the results obtained from the modal analysis of the bridge, and a good agreement was found. The SHM system was subjected to major upgrades later. Apaydın (2010) assessed the earthquake performance of the Second Bosphorus bridge and its retrofit investigations. Apaydın et al. (2012) investigated the bridge’s dynamic characteristics under heavy traffic and no-traffic conditions.

Apart from conventional wired systems, wireless SHM systems are becoming increasingly popular in recent times. After pioneering studies by Straser and Kiremidjian (1996, 1998), wireless SHM systems have undergone huge improvements in the last years (Lynch 2002; Grosse et al. 2004; Krüger et al. 2005), which were followed by several experimental validations (Lynch et al. 2006; Wang et al. 2006; Loh et al. 2007). The experimental results confirm that modern wireless SHM systems are feasible, reliable, and more cost-effective than traditional wired ones. These studies stimulated the investigation of a new wireless SHM platform instead of the traditional wired SHM system on the FSM bridge (Erdik and Apaydın 2007; Picozzi et al. 2010).

### Assessment of the seismic behaviour of the FSM bridge due to a low-intensity earthquake

**Fig. 7:**
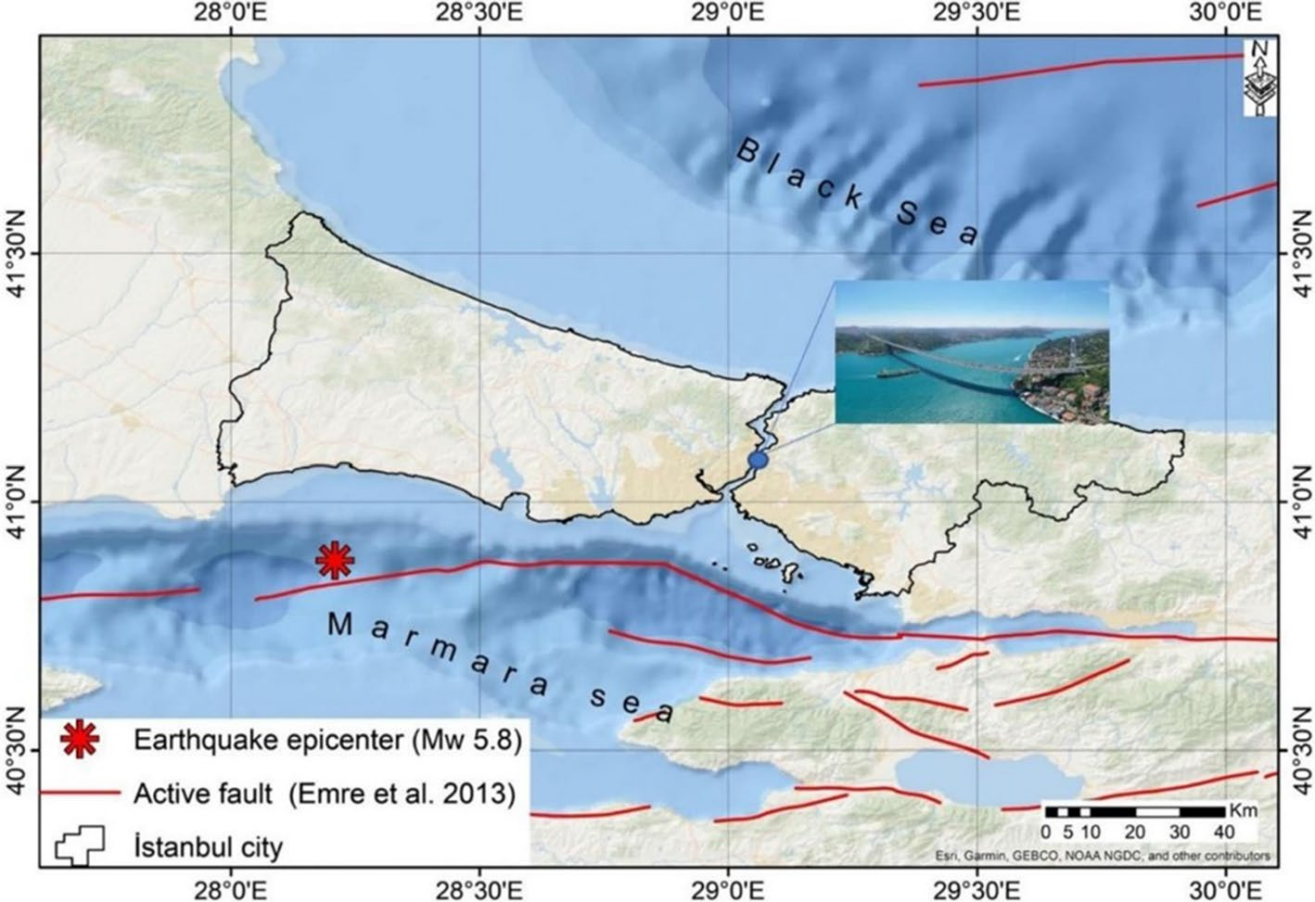
The FSM Bridge’s location and 2019 Silivri earthquake epicenter

On 26th September, 2019, an offshore event, the Silivri earthquake with a magnitude $M_w$ 5.8, occurred at a depth equal to 7 km on the Central Marmara Basin (CMB; Kumburgaz section) of the NAFZ at the coordinates 40.8823 N–28.2095 E as shown in Fig. 7. Although the earthquake was felt in a number of cities in the Marmara region, only minor damage was reported in a few Istanbul counties. However, the event was one of the region’s most significant strong ground motions since the 1999 Kocaeli and Düzce earthquakes, with magnitudes of $M_w$ 7.6 and $M_w$ 7.1, respectively (for which no data were available since, as mentioned, the bridge was first instrumented in 2001).

**Fig. 8:**
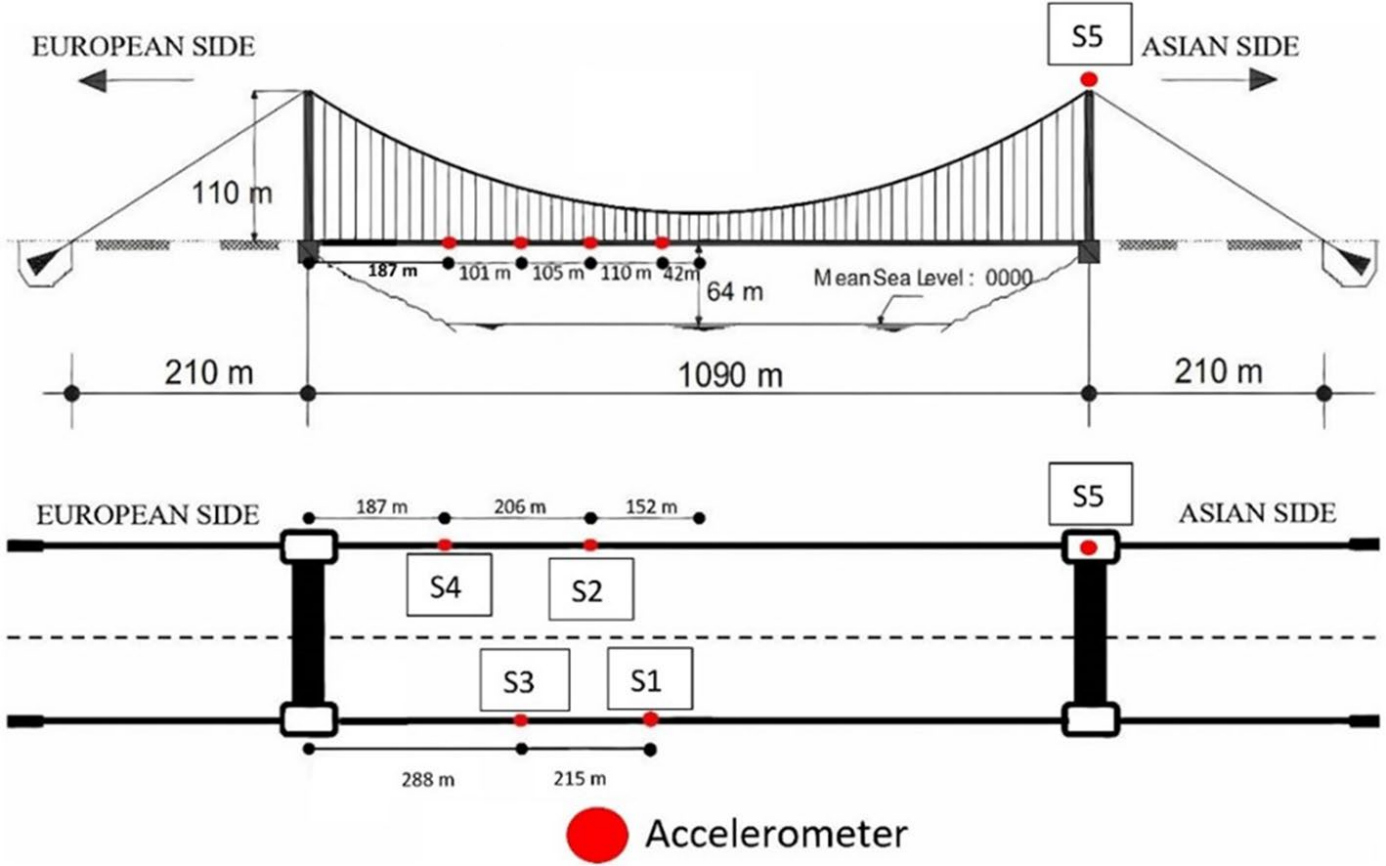
Sensors configuration on the deck of the FSM Bridge and location of the station used for the analysis

Data collected from the SHM system on the FSM Bridge during the Silivri earthquake are examined here. Figure 8 shows the sensor positions on deck and tower used to infer the bridge response recorded under the Silivri earthquake. The four stations on the deck (S1–S4) are placed at respectively 42, 152, 257, and 358 m away from the bridge’s mid-span towards the European side. The only recorded data for the towers’ stations is at the south tower (Asia Tower, S5), as depicted in Fig. 8. The stations on the deck recorded three components of the bridge response, namely the vertical, longitudinal, and transverse components of the motion. However, the vertical component of the motion was not recorded for station S5 on the south tower. The data sampling rate is 100 Hz. Table 3 lists the maximum recorded raw acceleration values at the five stations.

**Table 3:** Maximum recorded accelerations at the S1–S5 stations on the FSM Bridge during the Silivri earthquake (see Fig. 8)

Channel	Acceleration (cm/s^2^)				
	S1	S2	S3	S4	S5
Longitudinal	15.4837	12.5860	18.5386	21.2356	25.1690
Transverse	13.2349	11.7837	13.1889	13.8375	14.9203
Vertical	37.7262	28.8950	33.6714	39.7638	–

Since no damage was realistically expected due to this low-intensity seismic event, and mainly in order to point out some aspects of the whole assessment procedure, a basic analysis was performed by using the peak picking method on PSD functions to assess the dynamic characteristics of the bridge based on post-event ambient vibrations. Acceleration data collected from the aforementioned five stations (S1–S5) on the bridge (see Fig. 8), were used for the analyses. Raw acceleration data were pre-processed by detrending (baseline correction, DC component subtraction) and bandpass filtering techniques. A standard fourth-order Butterworth bandpass filter with 0.05 and 5.0 Hz corner frequencies was applied. The corner frequencies were determined according to the bridge’s modal frequencies as estimated by numerical analyses available in the literature (Apaydın et al. 2012).

The first 10 eigenfrequencies corresponding to the deck and towers were obtained from the recorded data and are compared with corresponding experimental results from previous ambient vibration studies in Table 4.

**Table 4:** Identification of the FSM Bridge: experimental results of the present work and comparison with ambient vibration results from the literature

Mode type	Mode shape	Frequency (Hz)			
		Dumanoglu et al. (1992)	Brownjohn et al. (1992a)	Picozzi et al. (2010)	Present study
Deck	1st TRsym	0.076	0.077	0.076	0.080
	1st Vasym Long	0.108	0.108	–	0.110
	2nd Vasym Long	0.125	0.125	0.122	0.125
	1st Vsym	0.145	0.155	0.152	0.155
	1st TRasym	0.159	0.208	0.207	0.205
Tower	2nd TRsym	0.211	0.221	0.220	0.220
	2nd TRasym Tw	0.232	0.239	0.241	0.230
	3rd TRsym Tw	0.243	0.244	0.247	0.245
Deck	TO	0.250	0.250	0.302	0.260
Tower	3rd TRsym Tw	0.266	0.266	0.314	0.270

*TRsym* transverse symmetric;* Vasym* vertical asymmetric;* Vsym* vertical symmetric;* TO* torsional;* Tw* tower;* Long* longitudinal

**Fig. 9:**
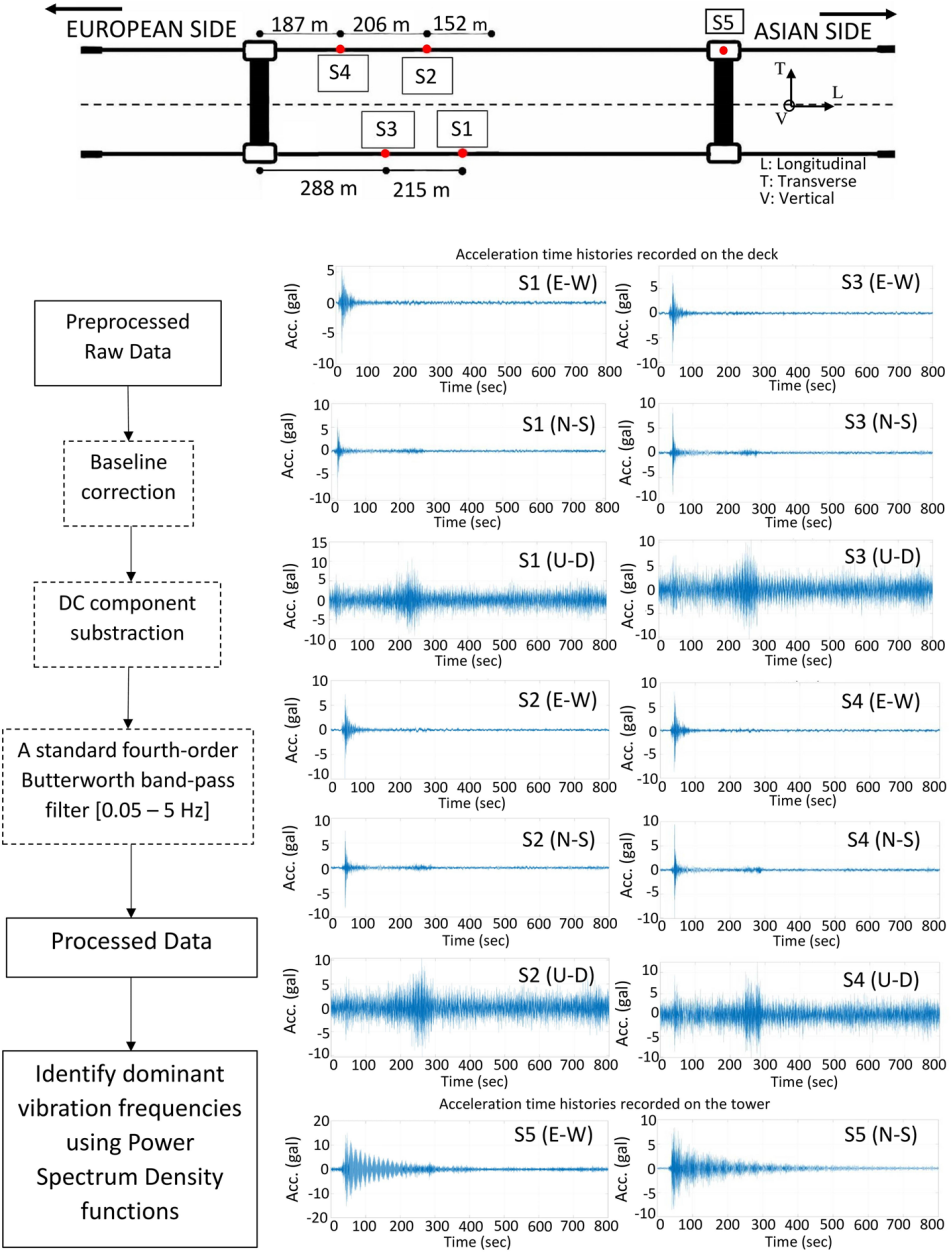
Recorded time histories on the bridge during the earthquake and the flow chart of the analysis

It is noted that ideally one should use for comparison the respective results from ambient vibration recordings just prior to the seismic event, so as to ensure that the various other environmental and operational factors affecting the response (e.g., wind, temperature, humidity, traffic conditions, aging, etc.) are essentially the same, however, such pre-event ambient data were unfortunately not available. Even with these limitations, it can be seen from Table 4 that the results of the current study are in general in good agreement with those of older analyses, and the possibility of damage to the bridge due to this low-intensity event is confirmed to be, as expected, rather insignificant (a fact also confirmed by later on-site inspections). The analysis procedure and the recorded time histories on the bridge during the earthquake are illustrated in Fig. 9.

It should once more be noted that several of the methodologies presented in the previous Sections could be used for more in-depth analyses, however this was beyond the scope of the current presentation. It should also be noted that a respective analysis of the bridge based on its recorded seismic response was not carried out, since, among others, this would require the knowledge of the base excitations, which were not available.

## Conclusions

This review work focuses on SHM methods applied for condition assessment of bridge systems. The overview focuses on schemes that target seismic evaluation and thus moves primarily within the domain of monitoring of dynamic behavior. The paper opens by overviewing available structural assessment schemes, making a distinction between sporadic (typically NDE-based) and continuous monitoring tools (usually referred to as SHM). Sections 2–4 focus on the latter category, offering a short review of the four levels of SHM, as identified in Rytter’s hierarchy of increasing complexity (Rytter 1993). Section 5 particularly focuses on the description of computational intelligence methods, defined as the pool of methods that draw from exploitation of evolutionary, swarm intelligence and neurocomputing algorithms. Use of these tools is overviewed for the tasks of optimal sensor placement, system identification and model updating, as well as continuous and vision-based health monitoring. Section 6 motivates the need for implementing SHM monitoring of large-scale bridge structures through the illustrative examples of two long-span suspension bridges in the Marmara Region in Turkey. A comprehensive overview of the SHM systems for these typical long-span suspension bridges is provided in detail. The general design considerations of the current and planned SHM systems, along with their project drawings of the geometrical properties of the bridges, are illustrated. Moreover, and mainly in order to point out some fundamental aspects of the damage assessment procedures, a basic analysis was performed to investigate the effects of a low-intensity seismic event at one of the bridges. The comparison of the ambient vibration results after the earthquake did not reveal significant deviations from earlier respective experimental results. Agreement of the results indicated that the bridge remained undamaged (a fact independently confirmed by field inspections). Considering the results gathered from this seismic event, it is apparent that for any seismic excitation in the future, the bridge’s health can be evaluated in almost real-time and thus provide the basis for rapid decision-making. In this, as well as all other important bridges, and depending on the instrumentation layout, the available data, the seismic intensity and the structural complexity, a variety of methodologies, as those presented in detail in the previous sections, are available for more in-depth structural health assessments after a seismic event, depending on the particular needs of the respective stakeholders.

## Data Availability

The raw/processed monitoring data required to reproduce the above findings cannot be shared at this time due to legal reasons.

## Abbreviations

ABC: Artificial bee colony
ANN: Artificial neural network
CMB: Central Marmara basin
DOF: Degree of freedom
EOP: Environmental operational parameter
FA: Firefly algorithm
FE: Finite element
FNN: Feed-forward neural network
FSM: Fatih Sultan Mehmet
GA: Genetic algorithm
GPS: Global positioning system
KGM: Turkish State Highways General Directorate
MA: Monkey search algorithm
MAC: Modal assurance criterion
MSC: Mode shape curvature
NAFZ: North Anatolian fault zone
NDE: Non-destructive evaluation
OMA: Operational modal analysis
OSP: Optimal sensor placement
PCA: Principal component analysis
PSD: Power spectral density
PSO: Particle swarm optimization
SHM: Structural health monitoring
TEM: Trans-European Motorway
